# METTL3-dependent m^6^A methylation facilitates uterine receptivity and female fertility via balancing estrogen and progesterone signaling

**DOI:** 10.1038/s41419-023-05866-1

**Published:** 2023-06-03

**Authors:** Shuo Wan, Yadong Sun, Jinbao Zong, Wanqing Meng, Jiacong Yan, Kexin Chen, Sanfeng Wang, Daji Guo, Zhiqiang Xiao, Qinghua Zhou, Zhinan Yin, Meixiang Yang

**Affiliations:** 1 The First Affiliated Hospital, Jinan University, Guangzhou, 510632 China; 2 The Biomedical Translational Research Institute, Guangzhou Key Laboratory for Germ-free animals and Microbiota Application, Key Laboratory of Ministry of Education for Viral Pathogenesis & Infection Prevention and Control, School of Medicine, Jinan University, Guangzhou, 510632 China; 3 Key Laboratory of Regenerative Medicine of the Ministry of Education, International Joint Laboratory for Embryonic Development and Prenatal Medicine, Department of Histology and Embryology, School of Medicine, Jinan University, Guangzhou, 510632 China; 4 Clinical Laboratory and Central Laboratory, the Affiliated Qingdao Hiser Hospital of Qingdao University, Qingdao, 266033 China; 5 Reproductive Medical Center, The First People’s Hospital of Yunnan Province, Kunming, 650021 China; 6 Guangdong Women and Children Hospital, Guangzhou, 510010 China; 7 Department of Neurology, Sun Yat-sen Memorial Hospital, 510123 Guangzhou, China; 8 Guangdong Provincial Key Laboratory of Tumor Interventional Diagnosis and Treatment, Zhuhai Institute of Translational Medicine, Zhuhai People’s Hospital Affiliated with Jinan University, Jinan University, Zhuhai, 519000 China

**Keywords:** Infertility, Experimental models of disease

## Abstract

Infertility is a worldwide reproductive health problem and there are still many unknown etiologies of infertility. In recent years, increasing evidence emerged and confirmed that epigenetic regulation played a leading role in reproduction. However, the function of m^6^A modification in infertility remains unknown. Here we report that METTL3-dependent m^6^A methylation plays an essential role in female fertility via balancing the estrogen and progesterone signaling. Analysis of GEO datasets reveal a significant downregulation of *METTL3* expression in the uterus of infertile women with endometriosis or recurrent implantation failure. Conditional deletion of *Mettl3* in female reproductive tract by using a *Pgr*-Cre driver results in infertility due to compromised uterine endometrium receptivity and decidualization. m^6^A-seq analysis of the uterus identifies the 3’UTR of several estrogen-responsive genes with METTL3-dependent m^6^A modification, like *Elf3* and *Celsr2*, whose mRNAs become more stable upon *Mettl3* depletion. However, the decreased expression levels of PR and its target genes, including *Myc*, in the endometrium of *Mettl3* cKO mice indicate a deficiency in progesterone responsiveness. In vitro, *Myc* overexpression could partially compensate for uterine decidualization failure caused by *Mettl3* deficiency. Collectively, this study reveals the role of METTL3-dependent m^6^A modification in female fertility and provides insight into the pathology of infertility and pregnancy management.

## Introduction

Infertility is a universal health issue, with an estimated 15% of couples experiencing infertility worldwide [1]. Endometrial disorders, including endometriosis and chronic inflammation, are the major reasons for female infertility. Endometriosis afflicts more than 10% of reproductive women, and those with stage III/IV endometriosis experience significantly lower rates of implantation and pregnancy [2, 3]. In addition, women with severe endometriosis have lower pregnancy rates compared with women with mild endometriosis [4]. Despite multiple IVF (in vitro fertilization) treatments, 10–15% of women still fail to achieve pregnancy, which is defined as recurrent implantation failure (RIF) [5]. The underlying mechanisms behind endometrial disorders remain unclear.

A growing body of evidence shows that epigenetic aberrations, including DNA methylation [6], histone acetylation [7], and noncoding RNAs [8, 9], might contribute to endometrial disorders. m^6^A, the most prevalent RNA modification in eukaryotes, plays important roles in RNA splicing, translocation, stability, and translation [10]. The functional effects of m^6^A are mediated by “writer”, “eraser”, and “reader” proteins [11]. The writer complex, consisting of a core METTL3-METTL14 m^6^A methyltransferase along with regulatory subunits, such as KIAA1429, RBM15, RBM15B, WTAP, and ZC3H13, catalyzes the m^6^A methylation of mRNA [10]. The eraser enzymes, including FTO and ALKBH5, mediate the reversal of this methylation. m^6^A methylated transcripts are recognized by reader proteins including the YTH family (YTHDF1/2/3 and YTHDC1/2), HNRNPA2/B1, HNRNPC, HNRNPG (RBMX), the insulin-like growth factor 2 mRNA-binding protein family IGF2BP1/2/3 [10]. Several studies have revealed the association of m^6^A modification with gametogenesis and fertility for both sexes [12, 13]. m^6^A modification has also been implicated in the pathogenesis of endometrium-related diseases. Reduced levels of m^6^A appeared to play an oncogenic role in patients with endometrial cancer by activating the AKT pathway [14]. Through extensive mining of public databases, Zhai et al. revealed that m^6^A levels are reduced in the endometrium and myometrium of women suffering from adenomyosis compared with endometrium from healthy candidates [15]. The dysregulation of m^6^A regulators has also been observed in endometriosis [16]. METTL3-dependent m^6^A is engaged in the maturation of primary microRNA126 mediated by DGCR8, which further increased the migration and invasion of endometrial stromal cells in endometriosis [17]. However, the function of m^6^A modification in infertility and uterine biology remains unknown.

Through extensive public database mining, we observed in this study that the mRNA of *METTL3* was decreased in endometrium from infertile women with endometriosis or RIF. Using uterine-specific *Mettl3*-deficient mice, we demonstrated that METTL3-mediated m^6^A modification is critical for implantation and decidualization. Mechanistically, loss of the m^6^A-modified sites might lead to estrogen dominance and progesterone resistance. We found that loss of *Mettl3* stabilizes the mRNA of estrogen target genes, such as *Elf3* and *Celsr2*, leading to hyperactivation of estrogen response. We also provide evidence in favor of METTL3 playing an important role in maintaining PR level and then driving c-Myc expression which is conducive to decidualization. Collectively, our findings contribute to the understanding of the etiology of female infertility, providing a molecular framework that might be useful for diagnostic and therapeutic strategies for infertility.

## Results

### METTL3 expression is reduced in endometrium of infertile women

**Fig. 1:**
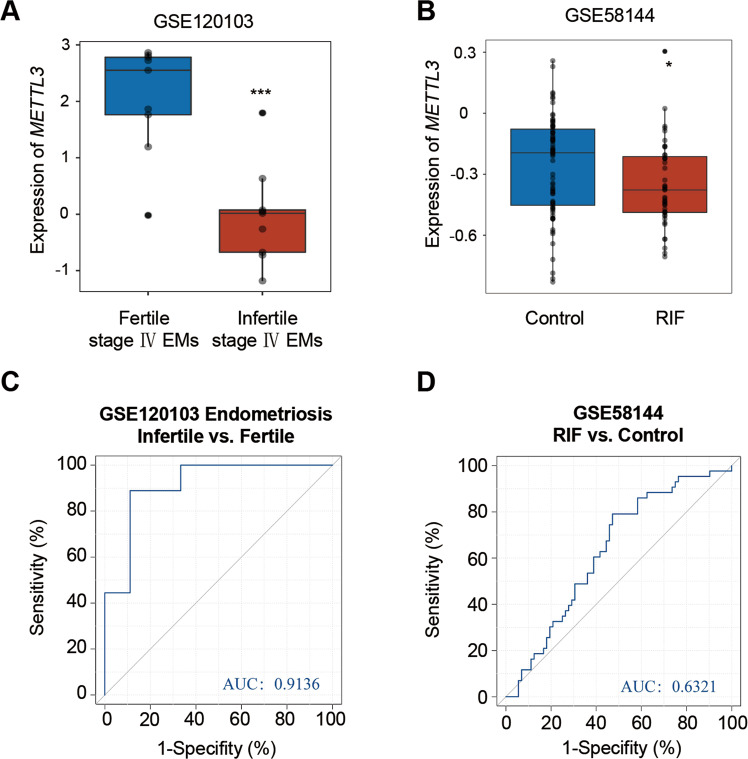
Expression landscape of *METTL3* in the endometrium of infertile women with endometriosis or recurrent implantation failure. **A** Expression landscape of *METTL3* in the endometrium of fertile (*n* = 9) and infertile (*n* = 9) women with stage IV endometriosis in dataset GSE120103. Data are presented as mean ± SD, ***P < 0.001, relative to “Fertile stage IV Endometriosis” group. **B** Expression landscape of *METTL3* in the endometrium of women with RIF following in vitro fertilization (IVF) treatment (*n* = 43) and healthy control women (*n* = 72) 7 days after the putative luteinizing hormone surge (GSE58144). Data are presented as mean ± SD, *P < 0.05, relative to control. ROC curve evaluation of the relationship between decreased expression of *METTL3* and infertile stage IV endometriosis (**C**), or RIF (**D**).

To address whether m^6^A regulators play a role in endometriosis-related infertility, we acquired gene expression profiles of endometrial tissue from the Gene Expression Omnibus (GEO) database. From the analysis of the expression level of m^6^A regulators in dataset GSE120103, which includes fertile women with stage IV endometriosis and infertile women with stage IV endometriosis, we found that most m^6^A regulators, including *METTL3, CBLL1, ALKBH5, FTO, YTHDC1, YTHDF2, IGF2BP2, HNRNPA2B1, HNRNPC*, and *LRPPRC*, were significantly downregulated, while *ZC3H13* and *IGF2BP1* were significantly upregulated in infertile group versus fertile group (Fig. 1A, and Supplementary Fig. 1A). From the analysis of the expression level of m^6^A regulators in the endometrium of women with RIF using dataset GSE58144, we found that *METTL3, YTHDC2, YTHDF3, HNRNPC*, and *FMR1* were significantly decreased, while *ZC3H13* were significantly increased in RIF patients compared with healthy controls (Fig. 1B, and Supplementary Fig. 1B). These results suggest that m^6^A regulators play an important role in both endometriosis-related infertility and RIF. Further analysis of the correlation between decreased *METTL3* expression and disease using receiver operating characteristic (ROC) curves demonstrated that the area under the curve (AUC) for reduced endometrial *METTL3* expression was 0.9136 (95% confidence interval (CI), 77.5% to 100%) in infertile patients with stage IV endometriosis (Fig. 1C), indicating downregulation of endometrial *METTL3* could well distinguish infertile from fertile endometriosis patients. Similarly, the AUC value for RIF was 0.6321 (95% CI, 53% to 73.5%) (Fig. 1D), indicating that reduced endometrial *METTL3* could well distinguish RIF patients from healthy controls. Collectively, these results suggested that the reduction of METTL3 might be associated with infertility.

### Ablation of *Mettl3* results in infertility due to embryo implantation failure

**Fig. 2:**
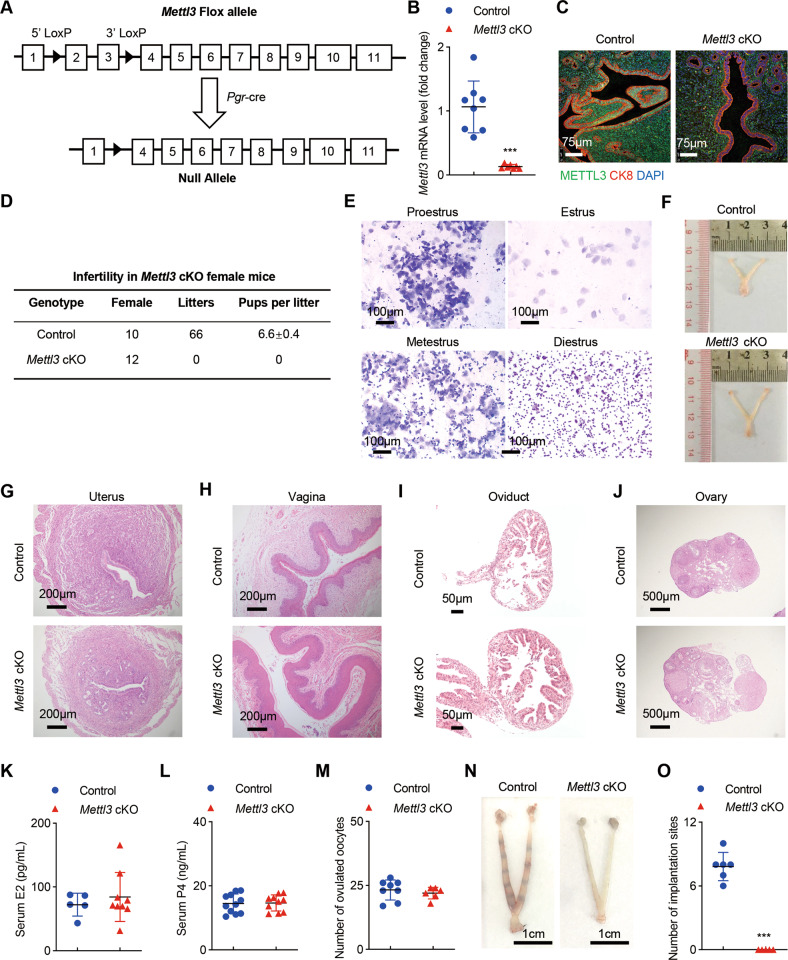
Uterine *Mettl3* deficiency induces complete implantation failure resulting in female infertility but displaying normal ovarian function. **A** Illustration of the *Mettl3* conditional allele with loxP sites placed flanking exon 2 and exon 3. **B** Relative *Mettl3* mRNA level in the uteri of *Mettl3* cKO (*n* = 7) and control (*n* = 8) mice on GD4. Data are presented as mean ± SD, ***P < 0.001, relative to control. **C** Immunofluorescence of METTL3 and CK8 in the uteri of *Mettl3* cKO and control mice on GD4. Nuclei were stained with DAPI. Scale bars: 75 μm. **D** Female fertility was assessed. Data are presented as mean ± SD. **E** Vaginal smear assays of *Mettl3* cKO mice confirmed each stage of the normal estrous cycle. Scale bars: 100 μm. **F** Gross morphology of female reproductive tracts in *Mettl3* cKO and control mice at 8 weeks of age. HE staining of the cross-sections of the uterus (**G**), vagina (**H**), oviduct (**I**), and ovary (**J**) in control and *Mettl3* cKO mice at 8 weeks of age. Serum concentrations of E2 (**K**) or P4 (**L**) in *Mettl3* cKO (*n* ≥ 9) and control mice (*n* ≥ 5) were analyzed and shown as mean ± SD. **M** Number of oocytes collected from superovulated *Mettl3* cKO (*n* = 6) and control females (*n* = 8). **N** Representative photographs of control uterus (*n* = 6) with implantation sites and *Mettl3 cKO* uterus (*n* = 5) without blue bands on GD5. Scale bars, 1 cm. **O** The number of implantation sites in (**N**) were counted and are reported as mean ± SD, ***P < 0.001, relative to control.

Mouse models allow us to study the sequence of events involved in the occurrence and progression of diseases. To study the role of METTL3 in the uterus during pregnancy, we generated a mouse model with conditional deletion of *Mettl3* in *Pgr*-positive cells (Fig. 2A). To do so, we started with mice carrying homozygous alleles of *Mettl3* with loxP sites placed in exon 2 and exon 3. *Mettl3*^flox/flox^ mice were mated to mice carrying a *Pgr*-Cre allele, in which Cre is knocked into the *Pgr* locus allowing Cre protein expression to be driven by the native *Pgr* promoter [18]. We produced *Mettl3*^flox/flox^*Pgr*^cre/+^ (*Mettl3* cKO) mice, eliminating the expression of *Mettl3* in tissues expressing the progesterone receptor (PR), i.e., epithelium, stroma, and myometrium of the uterus [18]. Ablation of *Mettl3* in the uterus was confirmed by reverse transcription-quantitative polymerase chain reaction (RT-qPCR) (Fig. 2B), and immunofluorescence (Fig. 2C). We observed vaginal plugs in *Mettl3* cKO mice indicating that mating behavior was normal in these females. Female fertility was assessed by mating *Mettl3* cKO and control females with wild-type (WT) males continuously for 6 months and tracking the number of litters and pups produced by each female. The control females were fertile (average number of pups/litter: 6.6 ± 0.4), whereas *Mettl3* cKO females were sterile and did not produce any pups during the 6 months mating trial (Fig. 2D). Four stages of mouse estrous cycle were observed in *Mettl3* cKO mice by vaginal smears (Fig. 2E). The examination of the reproductive duct in *Mettl3* cKO mice did not exhibit any significant alterations compared with the controls (Fig. 2F). Also, no significant histology differences were found in the uterus (Fig. 2G), vagina (Fig. 2H), ampullar oviduct (Fig. 2I), or ovary (Fig. 2J) of *Mettl3* cKO mice compared with the control mice. And serum levels of estradiol-17β (E2) and progesterone (P4) in *Mettl3*-deficient mice and control mice were comparable (Fig. 2K, L). Superovulation of 6-week-old mice showed no significant difference in the quantity of oocytes released between *Mettl3* cKO and control mice (Fig. 2M). These results suggest that infertility in *Mettl3* cKO mice is primarily due to a uterine functional defect.

Blastocyst implantation into the uterus is an essential step for the establishment of pregnancy. To identify the stage-specific failure of pregnancy in *Mettl3* cKO females, we subsequently analyzed the implantation status in *Mettl3* cKO females. Chicago Blue dye was injected to visualize the number and location of implanted embryos in the uterus on gestation day (GD) 5. The uterine horns of WT control mice had an average of 7.83 ± 0.99 implantation sites that appeared normally spaced per pregnant female, whereas *Mettl3* cKO mice had no grossly visible implantation sites on GD5 (Fig. 2N, O). These results clearly indicate that uterine METTL3 is indispensable for normal embryo implantation.

### *Mettl3* deficiency impairs uterine receptivity and decidualization

**Fig. 3:**
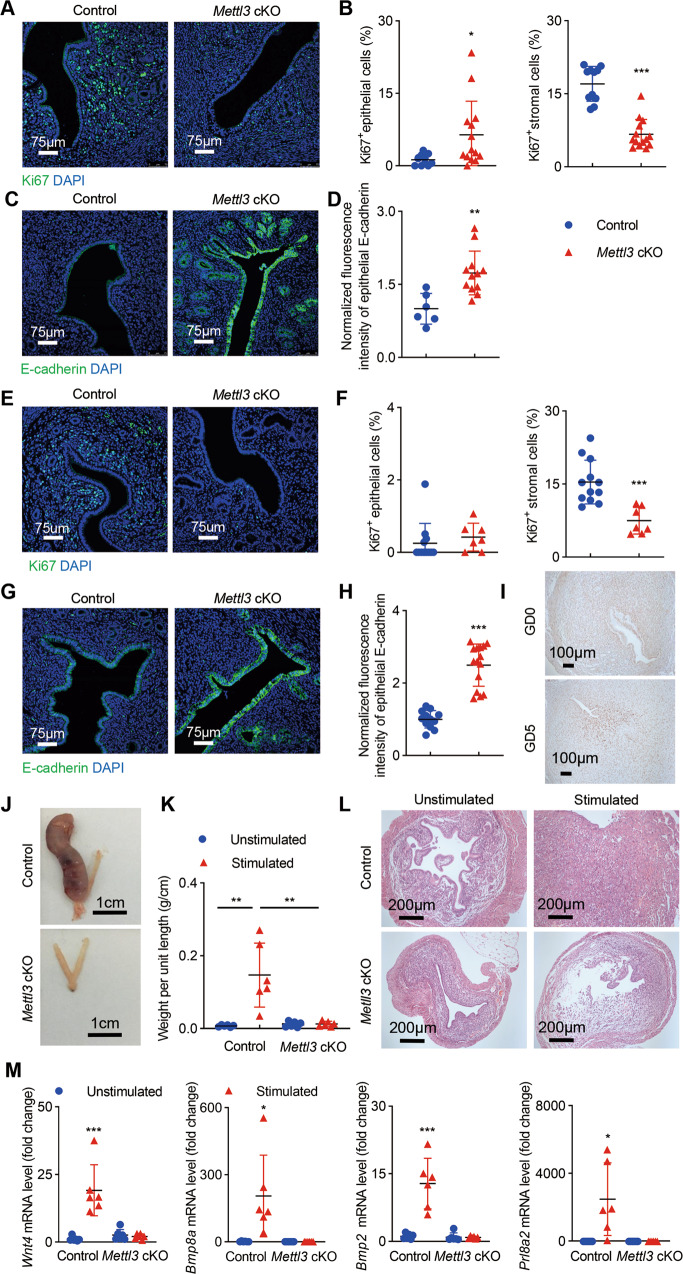
Ablation of *Mettl3* causes infertility due to compromised uterine receptivity and decidualization. Representative immunofluorescence images (**A**) and the percentage (**B**) of Ki67^+^ epithelial cells and stromal cells in uterine of *Mettl3* cKO and control mice on GD4. Data were calculated using 11 images from 3 control mice and 14 images from 3 *Mettl3* cKO mice. Representative immunofluorescence images (**C**) and quantification of E-cadherin (**D**) in uterine epithelial cells of *Mettl3* cKO and control mice on GD4. Fluorescence intensities of uterine epithelial E-cadherin were calculated using 6 images from 3 control mice and 12 images from 3 *Mettl3* cKO mice. Representative immunofluorescence images (**E**) and the percentage (**F**) of Ki67^+^ epithelial cells and stromal cells in uterine of *Mettl3* cKO and control mice following induction of artificial pregnancy (pollard experiment). Data were calculated using 12 images from 3 control mice and 7 images from 3 *Mettl3* cKO mice. Representative immunofluorescence images (**G**) and quantification of E-cadherin (**H**) in uterine epithelial cells of *Mettl3* cKO and control mice following induction of artificial pregnancy. Fluorescence intensities of uterine epithelial E-cadherin were calculated using 12 images from 3 control mice and 15 images from 3 *Mettl3* cKO mice. **A**, **C**, **E**, **G** Nuclei were stained with DAPI. Scale bars: 75 μm. **I** Immunohistochemistry of METTL3 with paraffin sections from pregnant females on GD0 and GD5. Sections are counterstained with hematoxylin. Brown staining denotes METTL3^+^ cells. Scale bars: 100 μm. **J** Representative pictures showing the gross morphology of oil-treated uterine horns (left horn) and untreated uterine horns (right horn) from control (*n* = 6) and *Mettl3* cKO mice (*n* = 6) collected 5 days after oil injection. Scale bars: 1 cm. **K** The weight per unit length of oil-injected and untreated uterine horns in (**J**). Data are presented as mean ± SD, **P < 0.01. **L** Histology of control and *Mettl3* cKO mice uterus in (**J**), as measured by HE staining, Scale bars 200 μm. **M** Relative mRNA levels of decidualization marker genes (*Wnt4*, *Bmp8a*, *Bmp2*, *Prl8a2*) in the uterine horns of control and *Mettl3* cKO mice in (**J**). **B**, **D**, **F**, **H**, **M** Data are presented as mean ± SD, *P < 0.05, **P < 0.01, ***P < 0.001, relative to control and unstimulated.

To reveal the underlying causes accounting for the defective implantation failure in *Mettl3* cKO mice, we investigated whether ablation of *Mettl3* alters uterine receptivity and decidualization. We first examined the proliferation versus differentiation status of uterine cells using Ki67 immunostaining at pre-implantation. As shown in Fig. 3A, in control mice, cell proliferation was reduced in epithelial cells before embryo attachment and increased in stromal cells in preparation for implantation on GD4 (Fig. 3A, B). However, the proliferative responses in stromal compartments of the uterus from *Mettl3* cKO mice were significantly reduced on GD4 compared with the control mice (Fig. 3A, B). E-cadherin, a cell polarity and cell junction marker, showed higher apical expression in *Mettl3* cKO mice compared with the controls (Fig. 3C, D). These observations collectively indicated abnormal uterine receptivity in *Mettl3* cKO mice in peri-implantation.

To further ascertain the role of METTL3 in uterine receptivity, E2 and P4 were administered to ovariectomized control and *Mettl3* cKO mice to mimic early pregnancy (pollard experiment) (Supplementary Fig. 2A). As expected, sequential E2 and P4 treatment induced uterine stromal cell proliferation in control mice (Fig. 3E, F). However, neither uterine stromal cells nor epithelial cells displayed a proliferative response in *Mettl3*-deficient mice (Fig. 3E, F). Increased E-cadherin expression was also detected in the luminal epithelium of *Mettl3* cKO mice compared with the controls in the pollard experiment (Fig. 3G, H). These data support the hypothesis that *Mettl3* deficiency causes endometrial receptivity abnormalities.

In response to implantation, stromal cells surrounding the mucosal crypt where the embryo resides proliferate extensively and differentiate into polyploid decidual cells. As METTL3 protein level in the uterus was substantially increased from GD0 to GD5 (Fig. 3I), we next examined the impact of *Mettl3* ablation on decidualization using an artificial decidualization model (Supplementary Fig. 2B). The control mice displayed a decidual uterine horn that responded well to the artificial induction. However, *Mettl3* ablation entirely preclude decidualization (Fig. 3J, K), which was further confirmed by histological analysis (Fig. 3L). We also observed a significant decrease in the expression of decidualization markers, including *Wnt4*, *Bmp8a*, *Bmp2*, and *Prl8a2* in the uterus of *Mettl3* cKO mice (Fig. 3M). Taken together, these data suggest that *Mettl3* cKO mice were sterile as a result of defective uterine receptivity and decidualization.

### Ablation of *Mettl3* results in an abnormal uterine transcriptome

**Fig. 4:**
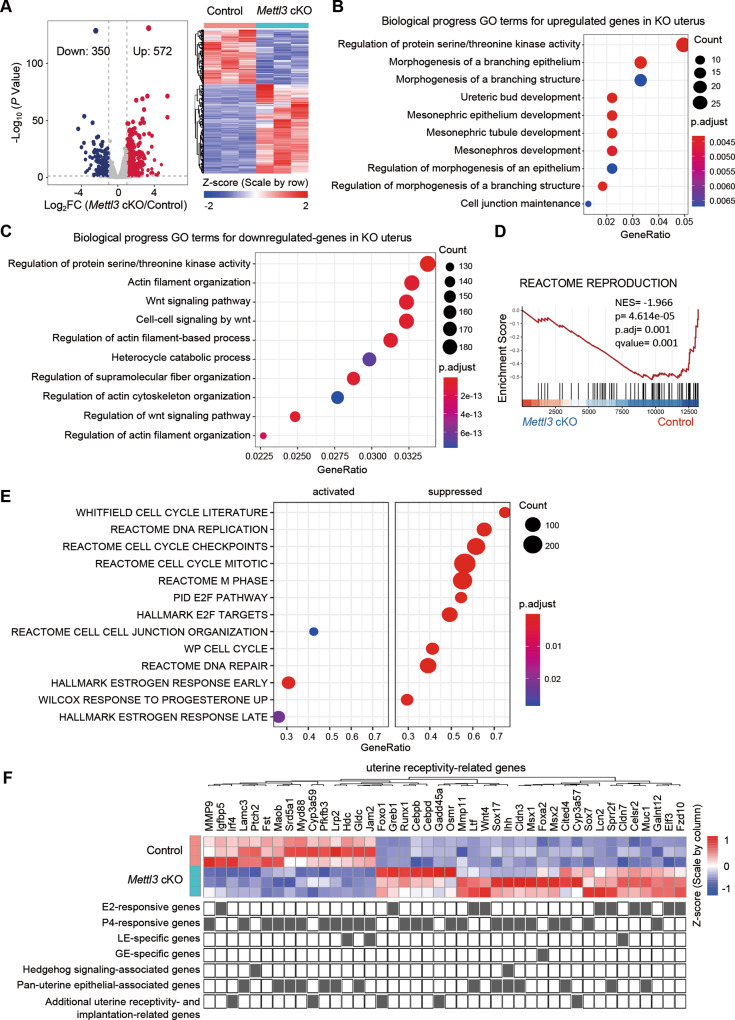
Transcriptome profile of the uteri in *Mettl3* cKO mice and control mice at the preimplantation stage. **A** Volcano plot depicting genes upregulated (red) or downregulated (blue) 1.5-fold or more in the uterus of *Mettl3* cKO mice vs control on GD4. A heatmap of differentially expressed genes is shown on the right (*n* = 3 mice per group). GO terms of the upregulated genes (**B**) and downregulated genes (**C**) in the uterus of *Mettl3* cKO mice compared with that of control mice. **D** GSEA of “REACTOME_REPRODUCTION” gene set in the uterus of *Mettl3* cKO mice relative to control mice. **E** GSEA analysis of the indicated gene sets in the uterus of *Mettl3* cKO mice relative to control mice**. F** Heatmap of differentially expressed uterine receptivity-related genes between *Mettl3* cKO and control mice on GD4 generated from RNA-seq data.

To understand the molecular basis of the implantation failure phenotype in *Mettl3* cKO mice, we performed an RNA-seq analysis of the uterine tissue of *Mettl3* cKO and control mice on GD4. Global gene expression profiles of *Mettl3* cKO versus control mice were analyzed. 922 genes were differentially expressed, including 572 up-regulated genes and 350 down-regulated genes in the uterus of *Mettl3* cKO mice compared with control mice (Fig. 4A). GO enrichment analysis showed that the up-regulated genes were mainly enriched in the regulation of epithelium, such as “Regulation of morphogenesis of an epithelium”, “Morphogenesis of a branching epithelium”, “Mesonephric epithelium development”, and “Cell junction maintenance” (Fig. 4B), while down-regulated genes were mainly involved in “Wnt signaling pathway”, “Regulation of actin filament-based process”, “Regulation of actin cytoskeleton organization” (Fig. 4C). GSEA analysis of MSigDB gene sets was performed (Fig. 4D, E). Specifically, the term “REACTOME REPRODUCTION” was observed negatively enriched in *Mettl3* cKO mice compared with the controls (Fig. 4D). And the term “REACTOME CELL CELL JUNCTION ORGANIZATION” was enriched in *Mettl3* cKO mice compared with the controls (Fig. 4E). These results suggested that METTL3 regulates luminal epithelial remodeling during the window of implantation, which is consistent with enhanced E-cadherin expression in the uterus of *Mettl3* cKO mice (Fig. 3C, G). Moreover, cell proliferation-related terms, including “WP CELL CYCLE”, “REACTOME DNA REPLICATION”, “HALLMARK E2F TARGETS” were suppressed in *Mettl3* cKO mice (Fig. 4E), which is consistent with the decreased proliferation in uterine stromal cells (Fig. 3A, E). Interestingly, among the significantly dysregulated genes, 42 genes associated with uterine receptivity and implantation were shown in the heatmap (Fig. 4F), including E2-responsive genes (*Igfbp5*, *Greb1*, *Ltf*, *Wnt4*, *Lcn2*, *Sprr2f, Celsr2*, *Muc1, Elf3*, *Fzd10*), P4-responsive genes (*Mmp9*, *Lamc3*, *Fst*, *Maob*, *Srd5a1*, *Myd88*, *Pfkfb3*, *Lrp2*, *Hdc*, *Gldc*, *Jam2*, *Runx1*, *Cebpb*, *Cebpd*, *Osmr*, *Mmp11*, *Sox17*, *Ihh*, *Cldn3*, *Msx1*, *Msx2*, *Cited4*, *Sox7*, *Galnt12*), LE-specific genes (*Hdc*, *Jam2*, *Cldn7*), GE-specific genes (*Foxa2*), hedgehog signaling-associated genes (*Ptch2*, *Ihh*), Pan-uterine epithelial-associated genes (*Lamc3*, *Maob*, *Srd5a1*, *Myd88*, *Pfkfb3*, *Lrp2*, *Gldc*, *Ltf*, *Sox17*, *Ihh*, *Cldn3*, *Cited4*, *Sprr2f*, *Muc1*), other uterine receptivity- and implantation-related genes (*Irf4*, *Cyp3a59*, *Foxo1*, *Gadd45a*, *Cyp3a57*). We believe that deregulated E2/P4-responsive genes in *Mettl3* cKO mice might be responsible for compromised uterine receptivity and decidualization.

### Loss of *Mettl3* leads to the activation of estrogen-driven transcriptional response by stabilizing mRNAs of the estrogen-regulated genes

**Fig. 5:**
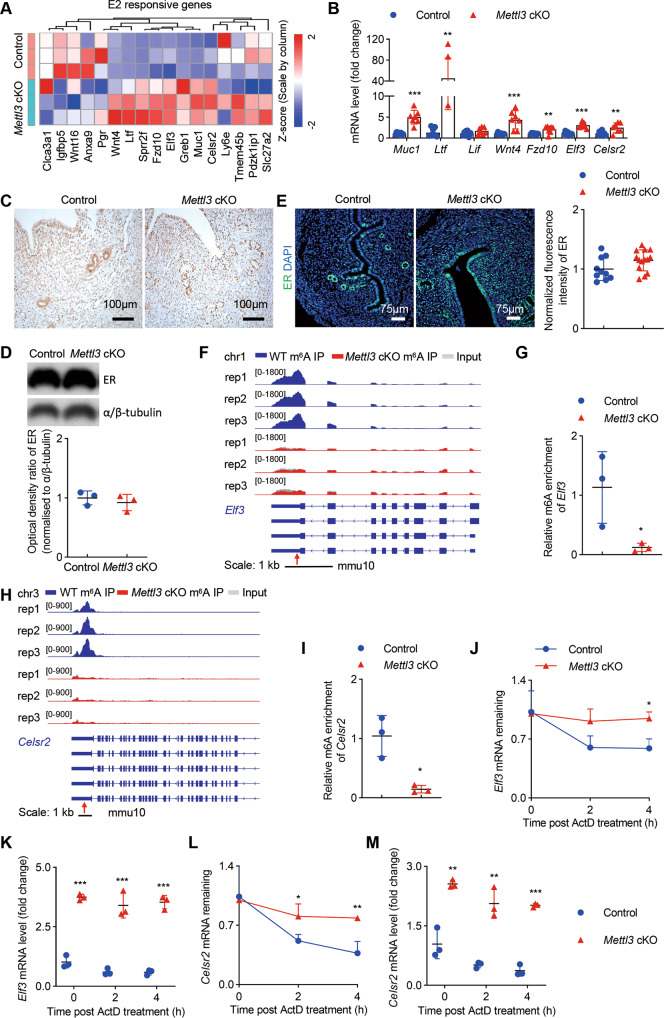
Loss of *Mettl3* leads to overactivation of estrogen signaling. **A** Heatmap of uterine E2-responsive genes in *Mettl3* cKO and control mice on GD4 generated from RNA-seq data. **B** Relative mRNA levels of E2-regulated genes in control (*n* = 8) and *Mettl3* cKO (*n* = 7) females on GD4. **C** Immunohistochemistry of hormone receptor ER with uterine sections from *Mettl3* cKO and control females on GD4. Sections are counterstained with hematoxylin. Brown staining denotes ER^+^ cells. Scale bars: 100 μm. **D** Immunoblotting analysis was conducted to compare uterine ER protein levels in control mice (*n* = 3) and *Mettl3* cKO mice (*n* = 3) on GD4. The experiments were repeated three times. α/β-tubulin was used as the loading control. Values are expressed as the mean ± SD. **E** Representative immunofluorescence images and quantification of ER in the uterus of *Mettl3* cKO and control mice following induction of artificial pregnancy. Nuclei were stained with DAPI. Scale bars: 75 μm. Fluorescence intensities of uterine ER were calculated using 10 images from 3 control mice and 14 images from 3 *Mettl3* cKO mice. Results are representative of 3 independent experiments. Integrative Genomics Viewer (IGV) tracks displaying transcripts and m^6^A peaks distribution of *Elf3* (**F**) and *Celsr2* (**H**) mRNAs in m^6^A-seq. The high-confidence m^6^A site is marked as an arrow. m^6^A enrichment in *Elf3* (**G**) and *Celsr2* (**I**) mRNA in the uterus of *Mettl3* cKO (*n* = 3) and control mice (*n* = 3), as determined by m^6^A-RIP-qPCR. RNA stability assay. The relative mRNA levels of *Elf3* (**J**) and *Celsr2* (**L**) were detected by RT-qPCR. The remaining mRNAs were normalized to *t* = 0. (*n* = 3 per group, biological repeated 3 times). RT-qPCR analysis of *Elf3* (**K**) and *Celsr2* (**M**) mRNA abundance. Relative expression was normalized to *t* = 0 of control uterine cells (*n* = 3 per group, biological repeated 3 times). Data are presented as mean ± SD, *P < 0.05, **P < 0.01, ***P < 0.001.

The implantation window in mouse uterus occurs between GD4 and GD5 and is characterized by a transition from an E2-dominant proliferative state to a P4-responsive state [19–21]. GSEA analysis revealed that “HALLMARK ESTROGEN RESPONSE EARLY”, “HALLMARK ESTROGEN RESPONSE LATE” were positively enriched in the uterus of *Mettl3* cKO mice (Fig. 4E). Concomitantly, the majority of E2 responsive genes were found to be upregulated in the uterus of *Mettl3* cKO mice compared with the controls (Fig. 5A). And RT-qPCR results further indicated that both E2-target epithelial genes (*Muc1*, *Ltf, Elf3, Celsr2*) and stromal genes (*Wnt4, Fzd10*) were significantly upregulated in the *Mettl3*-deficient uterus (Fig. 5B). We then measured uterine ER protein level in mice on GD4 using immunohistochemistry (Fig. 5C) and western blot (Fig. 5D) and in the pollard experiment using immunofluorescence staining (Fig. 5E), and found that uterine ER level in *Mettl3* cKO mice was not significantly altered compared with control mice (Fig. 5C–E).

To define the possible targets regulated by m^6^A modification involved in the functional maintenance of endometrial receptivity and female fertility, m^6^A-seq of the uteri of *Mettl3* cKO mice and the control mice were conducted. The abundance of m^6^A modifications in CDS, 3′UTR, 5′UTR, start codon, and stop codon were profiled (Supplementary Fig. 3A, B). After identifying the representative consensus of the m^6^A motif in the uterus of *Mettl3* cKO and control mice (Supplementary Fig. 3C), global hypomethylation of m^6^A at the transcription level (Supplementary Fig. 3D), we found that approximately 80% of methylated mRNAs contained 1 peak (Supplementary Fig. 3E). And we defined 319 hypo-methylated m^6^A genes whose mRNA transcripts were identified as down-regulated (*p* < 0.05, Hypo-down) and 1020 hypo-methylated m^6^A genes along with up-regulated mRNA transcript (*p* < 0.05, Hypo-up) in *Mettl3* cKO mice compared with WT controls (Supplementary Fig. 3F). Notably, the m^6^A level at 3’UTR of estrogen-responsive genes *Elf3* and *Celsr2* mRNA had substantially enriched m^6^A peaks in control uterus, but not in *Mettl3* cKO mice (Fig. 5F–I). To ascertain the role of METTL3-mediated m^6^A modification in regulating the mRNA levels of *Elf3* and *Celsr2*, mRNA decay assays were performed, and the results demonstrated that loss of m^6^A modification did appreciably inhibit the decay of *Elf3* and *Celsr2* mRNAs (Fig. 5J–M). Collectively, these results indicate that deficiency of METTL3-dependent m^6^A modification might increase the mRNA stability of E2-responsive genes, such as *Elf3* and *Celsr2*, leading to the overactivation of estrogen signaling in the uterus.

### *Mettl3*-deficient uteri show progesterone resistance due to reduced expression of PR and its downstream genes

**Fig. 6:**
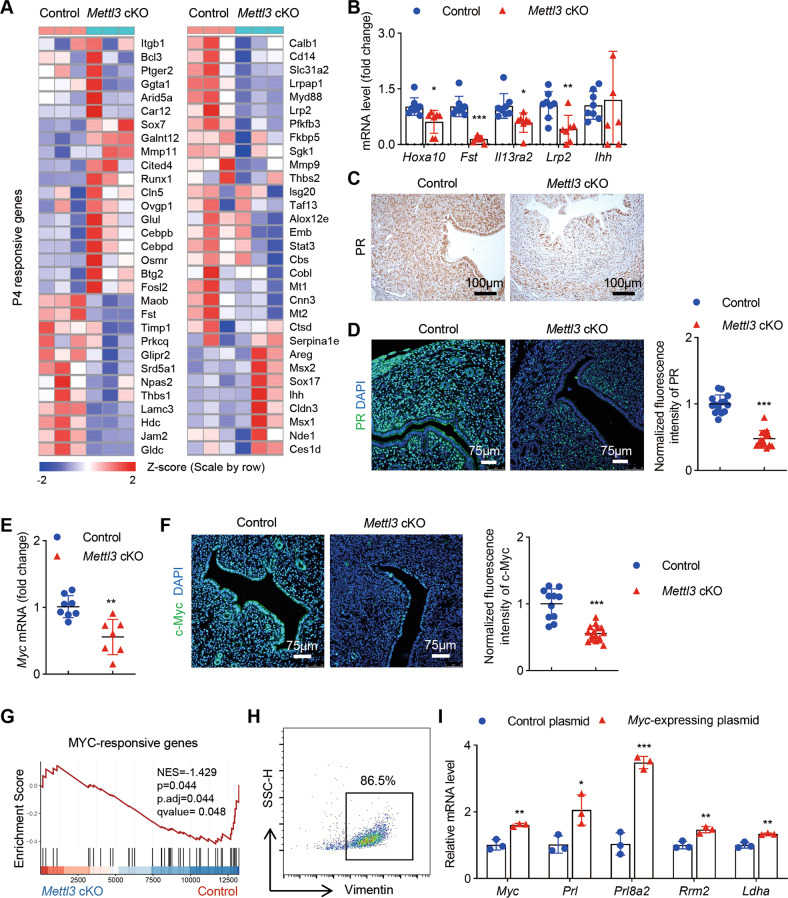
Progesterone resistance in *Mettl3*-deficient uteri could be due to reduced PR expression. **A** Heatmap of uterine P4-responsive genes in *Mettl3* cKO and control mice on GD4 generated from RNA-seq data. **B** Relative expression of mRNA for P4-regulated genes in control (*n* = 8) and *Mettl3* cKO (*n* = 7) females on GD4. **C** Immunohistochemistry of hormone receptor PR with uterine sections from *Mettl3* cKO and control females on GD4. Sections are counterstained with hematoxylin. Brown staining denotes PR^+^ cells. Scale bars: 100 μm. **D** Representative immunofluorescence images and quantification of PR in the uterus of *Mettl3* cKO and control mice following induction of artificial pregnancy. Nuclei were stained with DAPI. Scale bars: 75 μm. Fluorescence intensities of uterine PR were calculated using 14 images from 3 control mice and 15 images from 3 *Mettl3* cKO mice. **E** RT-qPCR of *Myc* mRNA expression in the uterus of control (n = 8) and *Mettl3* cKO mice (*n* = 7). **F** Representative immunofluorescence images and quantification of c-Myc in uterine epithelial and stromal cells of *Mettl3* cKO and control mice on GD4. Nuclei were stained with DAPI. Scale bars: 75 μm. Fluorescence intensities of uterine c-Myc were calculated using 11 images from 3 control mice and 16 images from 3 *Mettl3* cKO mice. Results are representative of 3 independent experiments. **G** GSEA analysis of “MYC-responsive genes” gene set in the uterus of *Mettl3* cKO mice relative to that in the uterus of control mice. **H** The isolated uterine stromal cells were stained with vimentin, and the purity was analyzed by flow cytometry. **I** RT-qPCR analysis was performed to examine the expression of *Myc*, *Prl*, *Prl8a2*, *Rrm2*, and *Ldha* in uterine stromal cells of *Mettl3* cKO mice after transfection of *Myc* overexpression plasmid or control plasmid for in vitro decidualization (biological repeated three times). Data are presented as mean ± SD, *P < 0.05, **P < 0.01, ***P < 0.001, relative to control.

The P4 and E2-responsive signaling pathways are tightly regulated in the endometrium. E2 drives uterine epithelial proliferation. During pre-implantation, E2 level is low, P4 initiates stromal cell proliferation, and primes the uterus to be receptive. P4 resistance and E2 dominance are most likely to happen when the balance between P4 and E2 signaling is lost [22]. Apart from overactivated estrogen signaling, *Mettl3* deficiency also hampers uterine P4 response. Most of the P4-responsive genes were found to be significantly downregulated in the uterus of *Mettl3* cKO mice as illustrated in Fig. 6A. Some of these genes were confirmed by RT-qPCR. The expression of P4-target molecules such as *Lrp2* in the epithelium and *Hoxa10*, *Fst*, and *Il13ra2* in the stroma was markedly downregulated in *Mettl3* cKO mice compared with the control (Fig. 6B). Furthermore, GSEA analysis revealed a negative enrichment of “WILCOX RESPONSE TO PROGESTERONE UP” pathway in the uterus of *Mettl3* cKO mice compared with their counterpart controls (Fig. 4E). Interestingly, although serum P4 levels were comparable in *Mettl3-*deficient mice and control mice (Fig. 2L), P4 and E2 co-treatment did induce uterine stromal cell proliferation in OVX WT control mice but not in *Mettl3* cKO mice (Fig. 3E), indicating that the loss of progesterone response in *Mettl3* cKO mice was not caused by decreased progesterone levels. P4 exerts its regulatory role by binding to its cognate receptor PR. To elucidate the possible reasons for progesterone resistance in *Mettl3* cKO mice, we examined the expression of uterine PR in GD4 pregnant mice (Fig. 6C) and in the pollard model (Fig. 6D), and found that the expression of PR was markedly decreased in the uteri of *Mettl3* cKO mice compared with the controls (Fig. 6C, D). Taken together, these observations suggest that METTL3 affects the endometrial response to progesterone during pregnancy by maintaining the expression level of PR.

To further confirm the repressed PR signaling in *Mettl3* cKO mice, we detected the expression level of PR target gene *Myc* [23]. c-Myc is a transcription factor involved in cell proliferation and is required for uterine stromal cell proliferation during peri-implantation [24–26]. We found that *Myc* mRNA and protein levels were significantly reduced in GD4 *Mettl3* cKO uterus compared with WT controls (Fig. 6E, F). We also noticed a decrease in the c-Myc protein level in the uterus of *Mettl3* cKO mice compared with control mice in the pollard model (Supplementary Fig. 4A). Using several MYC-target gene sets published previously [27, 28], GSEA revealed a significant decrease in MYC-target gene signatures in the uterus of *Mettl3-*deficient mice (Fig. 6G and Supplementary Fig. 4B, C), indicating *Mettl3* deficiency resulted in a compromised c-Myc signaling pathway. Furthermore, under in vitro decidualization, overexpression of *Myc* in *Mettl3*-deficient stromal cells could significantly upregulate the expression of reliable marker genes for decidualization, including *Prl*, *Prl8a2, Rrm2* [26] and *Ldha* [29] (Fig. 6H, I). These results indicated that *Mettl3*-deficient uteri show progesterone resistance due to reduced expression of PR and its downstream genes.

### The levels of P4 and E2-dependent genes *PR*, *MYC*, *ELF3* are correlated with *METTL3* in human endometrium

**Fig. 7:**
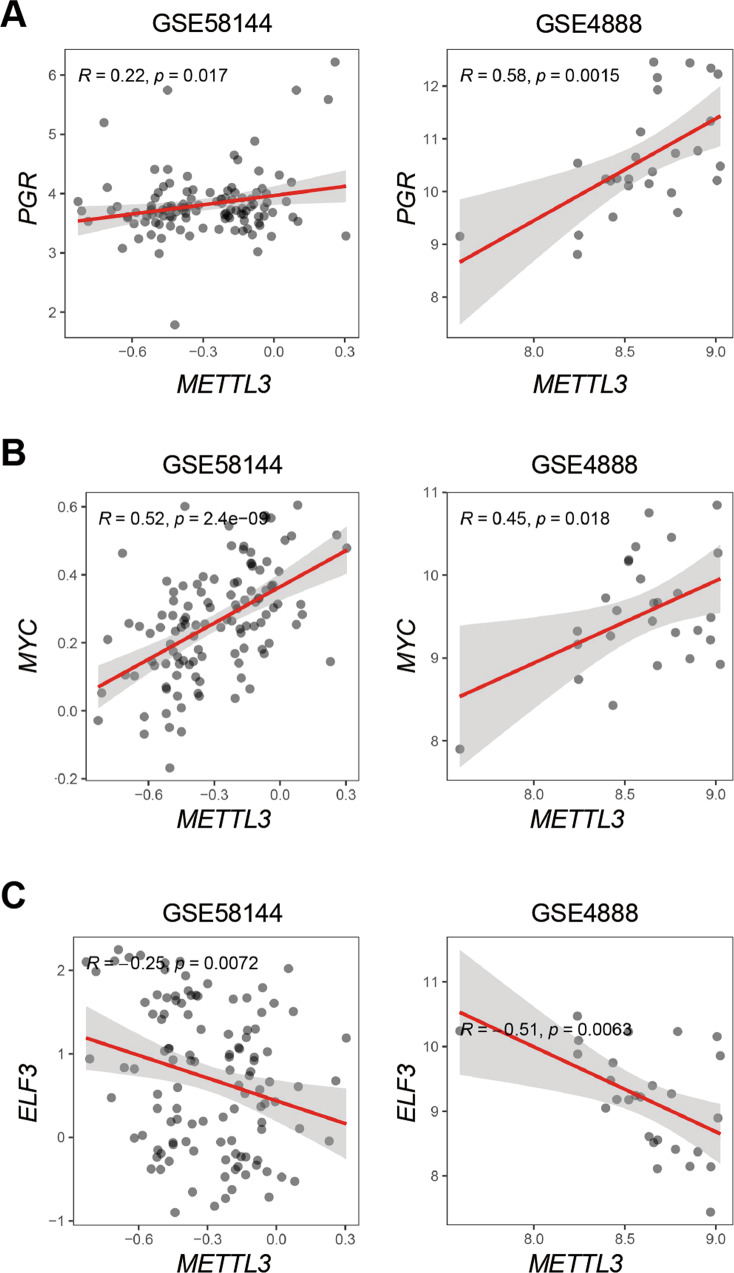
The levels of P4 and E2-dependent genes *PR*, *MYC, ELF3* are correlated with *METTL3* in human endometrium. **A** Pearson’s correlation of mRNA levels between *PGR* and *METTL3* in human endometrium (GSE58144 and GSE4888). **B** Pearson’s correlation of mRNA levels between *MYC* and *METTL3* in human endometrium (GSE58144 and GSE4888). **C** Pearson’s correlation of levels between *ELF3* and *METTL3* in human endometrium (GSE58144 and GSE4888).

To investigate whether METTL3 functions through regulating the balance between E2 and P4 signaling in human endometrium, we analyzed the correlation between *METTL3* and *PGR, MYC*, or *ELF3* in human endometrium from two independent cohorts, GSE58144 and GSE4888. *PGR* and *METTL3* mRNA levels were positively correlated in human endometrium (Fig. 7A), as well as the levels of *MYC* and *METTL3* (Fig. 7B). However, *ELF3* and *METTL3* mRNA levels were negatively correlated in human endometrium as indicated (Fig. 7C). The results above indicated that the expression of *PR*, *MYC*, *ELF3*, and *METTL3* is conserved between mouse and human. With this, a reasonable assumption might be that P4 resistance and E2 dominance could be related to the decrease of METTL3 in human endometrium in some disease conditions.

## Discussion

m^6^A methylation, regulating RNA stability, degradation, translation, alternative splicing, and gene expression, plays critical roles in various biological processes. m^6^A levels are elevated in the mouse uterus throughout pregnancy [30], suggesting m^6^A modifications are regulated by hormones. m^6^A demethylase FTO expression can be induced by estrogen in endometrial cancer via activation of the PI3K-Akt and MAPK pathways [31]. Our study reveals that *METTL3* expression is significantly decreased in endometrium from infertile women with endometriosis or recurrent implantation failure, suggesting that m^6^A modifications might be involved in the pathogenesis of infertility. However, the molecular mechanisms of METTL3 attenuation in the etiology and pathophysiology of infertility remain undetermined. Using *Mettl3* cKO mouse model, we provide evidence in favor that *Mettl3* loss causes infertility due to implantation failure.

The implantation window in mice occurs between GD4 and GD5, when the uterus becomes receptive [32–34], i.e., a reduction in the proliferation and polarity of uterine epithelial cells, as well as the induction of proliferation and decidualization of uterine stromal cells. E-cadherin, typically downregulated in the receptive epithelium, had stronger apical expression in the *Mettl3* cKO mice compared with the controls, indicating the abnormality of luminal epithelium differentiation during implantation. Meanwhile, persistent epithelial proliferation during the preimplantation stage results in implantation failure in several knockout mouse studies [35, 36]. In our study, a few of Ki67-positive cells were observed in the uterine epithelium of *Mettl3* cKO mice on GD4. Intriguingly, a significant reduction of Ki67-positive cells in the uterine stroma of the *Mettl3* cKO mice on GD4 was observed, indicating a defect in stromal cell proliferation in *Mettl3* cKO mice. Decidualization involves the differentiation of uterine stromal fibroblasts to decidual cells after embryo implantation, playing an important role in maintaining pregnancy. Decidualization defect of endometrial stromal cells has been regarded as a primary cause of endometriosis-related infertility [37, 38]. And our findings from decidualization experiments in *Mettl3* cKO mice support this notion.

A receptive uterus is characterized by a uterine transition from an E2-dominant proliferative state to a P4-responsive state [19–21]. To ascertain the role of METTL3 in E2 and P4 responses, we performed GSEA analysis based on MSigDB gene sets, and found that “HALLMARK ESTROGEN RESPONSE EARLY”, “HALLMARK ESTROGEN RESPONSE LATE” were positively enriched, whereas “WILCOX RESPONSE TO PROGESTERONE UP” was negatively enriched in *Mettl3* cKO mice compared with the controls. We also profiled the level of E2 and P4 responsive genes in the uterus on GD4, and found that most of the E2 responsive genes were sharply upregulated, while most P4 responsive genes were significantly decreased. These results established the critical role of METTL3 as a mediator of both estrogen and progesterone signaling in the uterus.

For the E2 responsive genes, we found that *Elf3* mRNA level is significantly increased in *Mettl3* cKO mice, and its m^6^A enrichment at 3′UTR is sharply decreased. Further mRNA decay assays demonstrated an increase in *Elf3* mRNA stability in the absence of METTL3-dependent m^6^A modification. ELF3 is an m^6^A reader, which can directly bind to the m^6^A site at the 5′UTR of mRNAs participating in the translation initiation of specific genes. In addition, ELF3 binds to ERα in the absence of E2, but dissociates with ERα upon E2 treatment in a dose- and time-dependent manner [39], suggesting a possible feedback loop between ERα signaling and ELF3. Another E2-responsive gene *CELSR2* is negatively associated with the overall survival time of endometrial cancer patients [40]. *CELSR2* deficiency impaired cell proliferation of hepatocytes [41], liver cancer cells [42], and Schwann cells [43]. Interestingly, uterine *Celsr2* in *Mettl3* cKO mice reflects a similar mRNA expression and m^6^A modification pattern to *Elf3*. Based on our mRNA decay assays, deficiency of METTL3-dependent m^6^A might increase the mRNA stability of *Celsr2*.

E2 and P4 play a regulatory role by binding to their cognate receptors, ER and PR, respectively. Moreover, the level of hormone receptors can be regulated by m^6^A modification. ERα mRNA m^6^A methylation is significantly upregulated by R-2HG due to FTO degradation, which may then result in suppressing ERα protein expression via translational regulation, hence reducing cholangiocarcinoma [44]. The elevated Estrogen Related Receptor γ in chemoresistant cancer cells can be attributed to m^6^A-dependent splicing of precursor *ESRRG* mRNA [45]. However, we did not observe METTL3-dependent m^6^A methylation in *Esr1*, *Esr2*, *Esrra*, and *Esrrb* mRNAs in mouse uterus (data not shown). In note, uterine ER level was not altered in *Mettl3* cKO mice compared with the controls. Endometrial stromal PR is mediated by E2/ER signaling [46]. And prior to conceptus implantation, PR expression is downregulated in the uterine epithelial compartment and is upregulated in uterine stromal cells [47]. Insufficient PR signaling hampers intimate stromal-epithelial crosstalk and thus uterine receptivity [48, 49]. We also found a sharp reduction of uterine stromal PR protein level in *Mettl3* cKO mice on GD4, and in the pollard experiment, when compared with the control mice. The results above indicated that METTL3 may regulate the P4 response of uterine stroma during pregnancy by upregulating PR.

ChIP-seq analysis identified PR-binding sites within *Myc* in the uterus of mice treated with P4 [23], implying that c-Myc is a target of PR. The Myc family proteins contain three well-defined members: c-Myc, N-Myc, and L-Myc. NDRG2 [50] and NDRG4 [51], downstream-regulated genes of N-Myc, are upregulated at implantation sites during early pregnancy in mice, and their downregulation inhibit the decidualization process of mouse endometrial stromal cells. c-Myc, a well-known oncoprotein, is a transcription factor involved in ribosome biogenesis, protein translation, cell-cycle progression, and metabolism, orchestrating a broad range of biological functions, such as cell proliferation, and differentiation [52]. On day 4 of pregnancy, an increase in c-Myc expression in uterine stromal cells was identified, accompanied by a robust proliferation of stromal cells [24]. E2 administration raised epithelial c-Myc levels and DNA synthesis rapidly [24, 25], and P4 administration elevated stromal c-Myc levels in vivo and in vitro [24, 26]. We detected a decrease in the protein levels of c-Myc in the uterus of *Mettl3* cKO mice, and *Myc* overexpression was able to partially restore the deficit of decidualization in vitro, further confirming that METTL3 may regulate the hormone response of uterine epithelial and stromal cells by influencing the expression of PR and its target genes.

In summary, this study provides evidence in favor of the critical role of METTL3-dependent m^6^A methylation in maintaining a balanced estrogen and progesterone signaling pathway, which is conducive to endometrial receptivity and female fertility, thereby providing insightful information for the pathology of infertility and pregnancy management.

## Materials and methods

### GEO data processing

RIF gene expression profiles (GSE58144) and endometriosis gene expression profiles (GSE120103, GSE4888) with clinical information were downloaded from GEO database (https://www.ncbi.nlm.nih.gov/geo/) using “GEOquery” package (v2.64.2) in R software (v4.2.1). For data processing, the “limma” package (v3.52.2) was applied for background correction and quantile normalization of all the raw data files, and the expression values were then obtained. The probe set with max-average value was chosen as the expression value for the same gene with multiple probe sets. Barplots, ROC curves, and correlation analysis were drawn using “ggplot2” package (v3.4.0). The code used for GEO data processing is available at https://github.com/wansh007/data-process.

### Mice

C57BL/6N-*Mettl3*^em1cyagen^ (*Mettl3*^flox/flox^) mice were generated by Cyagen Biosciences Inc (Guangzhou, China) using the CRISPR–Cas9-based genome-editing system. *Pgr-Cre* mice were obtained from Cyagen Biosciences Inc (Guangzhou, China). To generate *Mettl3* cKO mice, *Mettl3*^flox/flox^ mice were bred to mice carrying the *Pgr-Cre* knock-in allele to obtain *Mettl3*^flox/flox^ *Pgr*^Cre/+^ mice (*Mettl3* cKO mice). The females were chosen for the indicated experiments. To reduce the effect of genetic background variability, littermate floxed and gene-deleted mice were used in the same experiments. All mice used in this study were housed in specific pathogen-free animal facilities in the Animal Resource Center at Jinan University, following the ethical guidelines of the Animal Ethics Committee of Jinan University (IACUC-20220418-03).

### HE, immunofluorescence and immunohistochemistry staining

Uterine tissues were fixed in Paraformaldehyde Fix Solution (G1101-500ML, Servicebio) for 24 h and embedded in paraffin, and sectioned at 5 μm. Sections were dried at 60 °C for 30 min and stored at room temperature. Sections were deparaffinized and rehydrated in xylene, 100% ethanol, 90% ethanol, 85% ethanol, 75% ethanol and double distilled water, before HE, immunofluorescence and immunohistochemistry staining.

For HE staining, sections were stained with eosin (G1100, Solarbio) and hematoxylin (G1140, Solarbio). Images were captured using an Olympus BX53 microscope.

For immunofluorescence staining, antigen retrieval was carried out by heating the sections in sodium citrate buffer (10 mM sodium citrate, 0.05% Tween 20, pH 6.0) at 95 °C for 15 min. The sections were immersed in 3% H_2_O_2_ for 45 min at room temperature to quench endogenous peroxidase, permeabilized in 0.2% Triton-100 in PBS for 45 min, and then blocked with 1% (w/v) BSA Fraction V (ST023, Beyotime) and 10% goat serum (v/v) (B900780, Proteintech) in PBS before the primary antibodies were added. Then the sections were incubated with the following primary antibodies against METTL3 (ab195352, Abcam), CK8 (DSHB, TROMA-I), Ki67 (ab15580, Abcam), E-Cadherin (3195S, Cell Signaling Technology), PR (8757 S, Cell Signaling Technology), ER-alpha (ab32063, Abcam), c-MYC (1:200, 10828-1-AP, Proteintech) overnight at 4 °C. Followed by incubation with the secondary antibodies: Alexa Fluor 488-conjugated affiniPure Goat anti-Rabbit IgG (H + L) (1:400, 115-585-146, Jackson ImmunoResearch), Alexa Fluor 594-conjugated affiniPure Donkey Anti-Rat IgG (H + L) (1:400, 712-585-153, Jackson ImmunoResearch), Alexa Fluor 594-conjugated affiniPure Goat anti-Mouse IgG (H + L) (1:400, 111-545-144, Jackson ImmunoResearch) for 1 h, and nuclei-staining with DAPI (1:1000, D9542, Sigma) for 10 min at room temperature. Images were taken using a Leica TCS SP8 confocal microscope.

For immunohistochemistry, antigen retrieval was performed and endogenous peroxide was blocked as aforementioned. After being blocked with 1% (w/v) BSA Fraction V (ST023, Beyotime) and 10% goat serum (v/v) (B900780, Proteintech) in PBS for 1 h, the primary antibodies were added. The sections were incubated with the following primary antibodies against METTL3 (1:800, ab195352, Abcam), PR (1:400, 9856S, Cell Signaling Technology), ER-alpha (1:400, ab32063, Abcam), Ki67 (1:2000, ab15580, Abcam) overnight at 4 °C. On the following day, the sections were incubated with biotinylated goat anti-Rabbit IgG (1:400, BA-1000-1.5, Vector Laboratories) for 1 h at room temperature. After several rinses in PBS, the sections were incubated with the Vectastain Elite ABC reagent (PK-6100, Vector Laboratories) for 30 min, and immunoreactive signals were developed using ImmPACT DAB EqV Peroxidase (HRP) Substrate (SK-4103, Vector Laboratories), and counterstained with hematoxylin. Images were captured using an Olympus BX53 microscope.

Immunofluorescence images were analyzed using ImageJ (National Institutes of Health, v1.8.0_345). The level of E-Cadherin in uterine epithelial cells was determined by calculating the mean intensity of E-Cadherin signals. Similarly, the levels of ER, PR, and c-Myc in the uteri were determined by calculating the mean intensities of ER, PR, and c-Myc signals. The number of Ki67-positive (Ki67^+^) cells and the total number of epithelial or stromal cells were manually counted in several uterine sections from three mice in each group. Percentage of Ki67^+^ epithelial cells and percentage of Ki67^+^ epithelial cells were then calculated.

### Protein extraction and western blot analysis

Protein extraction and western blot analysis were performed as previously described [53]. Western blotting experiments were analyzed using the following antibodies against ER-alpha (1:1000, ab32063, Abcam), α/β-Tubulin (1:4000, 2148S, Cell Signaling Techology), goat anti-rabbit IgG-HRP (1:2500, AS006, Asbio Technology). The experiments were performed with 3 replicates.

### Fertility analyses

Female mice at least 8 weeks old were mated with fertile wild-type males to induce pregnancy (vaginal plug = day 1 of pregnancy). Uteri on day 4 post-mating were collected fixed in Paraformaldehyde Fix Solution (G1101-500ML, Servicebio) for histology or flash-frozen for RT-qPCR analysis. Successful pregnancy was confirmed by flushing embryos from the uteri on GD4. Tail intravenous injection with 0.1 mL of 1% Chicago blue dye (C8679, Sigma-Aldrich) was applied to identify implantation sites on GD5. Female fertility was assessed by mating cohorts of *Mettl3* cKO (*n* = 12) and control (*n* = 10) mice individually with WT males proven breeders continuously for 6 months. The numbers of pups per litter per dam were recorded as mean ± SD.

### Crystal violet staining of vaginal smear for mouse estrous cycle staging identification

The estrous cycle phases of *Mettl3* cKO and control mice (8–10 week) were determined by crystal violet staining of vaginal smears, as previously described [54]. Briefly, the vaginal cells were washed with 100 μL PBS and transferred to a dry glass slide using a pipette. The slide was air-dried and stained for 1 min with 1% crystal violet (V5265-500ML, Sigma), followed by three 1-min rinses in water. The slides were coated with neutral balsam (G8590-100ml, Solarbio) and viewed with an Olympus BX53 microscope.

### Measurement of serum estradiol and progesterone levels

Mouse blood samples were collected on GD4 in the morning and serum progesterone (P4), as well as estradiol-17β (E2) levels, were measured by Estradiol 2 Assay Kit (H102-1, NanJingJianCheng Bioengineering Institute, Nanjing, China) and Progesterone Assay Kit (H089, NanJingJianCheng Bioengineering Institute, Nanjing, China) according to the manufacturer’s recommendations.

### Hormone treatments

Mice were ovariectomized at 6 weeks of age and rested for 2 weeks to remove endogenous ovarian hormone in most experiments. For evaluating the effects of E2, a subcutaneous injection of 100 ng E2 (E8875, Sigma) was given to OVX mice and sacrificed 24 h after the last injection. The hormonal profile of pregnancy at the time of implantation was simulated using a “pollard” experiment scheme [55]. Briefly, mice were treated with daily subcutaneous injections of E2 (100 ng) for 2 days. Following this treatment and after 2 days of rest, the mice received a daily injection of P4 (1 mg) for 3 days. On the fourth day, mice were treated with 100 ng of E2 and 1 mg of P4. The mice were sacrificed 16 h after the E2 + P4 injection. The uteri were harvested for RT-qPCR and histology analysis.

### Superovulation

Superovulation studies were conducted to assess ovarian function. To induce superovulation, 6-week-old mice were administrated 7.5 IU of pregnant mare serum gonadotropin (PMSG, hor-272, ProSpec). Human chorionic gonadotropin (hCG, 230734, sigma) (7.5 IU) was injected subcutaneously 48 h after PMSG injection. At 14 h post hCG injection, the ovaries and oviducts were surgically removed, and the cumulus-oocyte complexes mass was recovered from the oviduct and collected into M2 medium (Sigma) containing 1 mg/mL of hyaluronidase (H3506, Sigma) to dissociate the cumulus cells from oocytes. The numbers of oocytes were counted and recorded.

### In vivo decidualization assay

In vivo artificial decidual response was conducted similarly to that described previously [56]. Briefly, the ovariectomized mice were given daily subcutaneous injections of 100 ng of E2 (E8875, Sigma) prepared in sesame oil for 3 days (day 1–3). On day 6–8, mice were given a subcutaneous injection of 1 mg of P4 and 6.7 ng of E2 dissolved in sesame oil. Artificial decidualization was processed by intraluminal injection of 50 μL of sesame oil into the right uterine horn 6 h after the last injection of E2 and P4, with the left uterine horn acting as a negative control. Mice received daily E2 and P4 administration for 5 more days and sacrificed on day 14.

### Isolation and culture of uterine cells

Uterine cells were isolated from day 4 pseudopregnant mice as previously described [57]. The uterine horns of control and *Mettl3* cKO mice were cut longitudinally, washed with HBSS, and digested with 1% (w/v) pancreatin (P7545, Sigma) and 6 mg/ml dispase II (Sigma) in HBSS for 1 h at 4 °C followed by 1 h at room temperature and 10 min at 37 °C. The tissues were rinsed with HBSS for three times, and the supernatant was filtered through a 100-μm nylon cell strainer and collected as epithelial cells. And the remaining tissues were further digested within 0.15 mg/ml collagenase I (17100017, Invitrogen) in HBSS at 37 °C for 30 min. The tissues were washed 3 times with HBSS. And the supernatant above was filtered through a 100-μm nylon cell strainer for the collection of uterine stromal cells. The uterine cells were cultured in DMEM/F12 (319-080-CL, Wisentbio) containing 2% charcoal-stripped FBS (04-201-1A, Biological Industries) and 15 mM HEPES (15630080, Thermo Fisher).

### In vitro decidualization assay

To induce stromal cells to undergo decidualization, the harvested stromal cells were cultured for 30 min, the medium was changed to remove unattached cells. And the cell culture was continued by adding fresh medium supplemented with P4 (1 μM) and E2 (10 nM) dissolved in ethanol for different time points. Transfection of *Myc* overexpression plasmid in *Mettl3* cKO uterine stromal cells was performed according to Lipo3000 protocol (L3000015, Life Technologies). For the six-well culture plate, 2 μg *Myc* overexpression plasmid (EX-Mm30812-M02, GeneCopoeia) or 2 μg control plasmid (EX-NEG-M02-B, GeneCopoeia) was used for the transfection. The cells were cultured with P4 (1 μM) and E2 (10 nM) for 2 days, and the mRNA levels of *Myc* and of decidualization-related genes were analyzed by RT-qPCR.

### RNA extraction and RT-qPCR analysis

Total RNA was extracted from uterine tissues using TRIzol reagent (Invitrogen, Carlsbad, CA, USA) according to the manufacturer’s protocol. The complementary DNAs (cDNAs) were synthesized with PrimeScript RT Master Mix (RR036A, Takara) by using 500 ng total RNAs according to the manufacturer’s instructions. Quantitative real-time PCR was performed to assess the expression of genes of interest with SYBR Green (RR820A, Takara) on a CFX Connect Real-Time PCR Detection System (Biorad). Experimental gene expression data were normalized to *Actb*. The RT-qPCR primers are listed in Table 1.

**Table 1:** List of primer sequences for RT-qPCR.

Primer name	Sequences (5′-3′)
*Mettl3* Forward	AACATCTGTGGCCCCTGAAC
*Mettl3* Reversed	TGGCAAGACGGATGGAAACA
*Wnt4* Forward	AGACGTGCGAGAAACTCAAAG
*Wnt4* Reversed	GGAACTGGTATTGGCACTCCT
*Bmp8a* Forward	CCTGGTCATGAGCTTCGTCA
*Bmp8a* Reversed	AGCAGGGATCTGGGTTAGGT
*Bmp2* Forward	TGCTTCTTAGACGGACTGCG
*Bmp2* Reversed	CTGGGGAAGCAGCAACACTA
*Muc1* Forward	GGCATTCGGGCTCCTTTCTT
*Muc1* Reversed	TGGAGTGGTAGTCGATGCTAAG
*Ltf* Forward	TGAGGCCCTTGGACTCTGT
*Ltf* Reversed	ACCCACTTTTCTCATCTCGTTC
*Lif* Forward	ATTGTGCCCTTACTGCTGCTG
*Lif* Reversed	GCCAGTTGATTCTTGATCTGGT
*Wnt4* Forward	GAGAACTGGAGAAGTGTGGCTG
*Wnt4* Reversed	CTGTGAGAAGGCTACGCCATAG
*Fzd10* Forward	CTGGCTTGCTACCTAGTCATCG
*Fzd10* Reversed	TGCGTACCATGAGCTTCTCCAG
*Hoxa10* Forward	GGCAGTTCCAAAGGCGAAAAT
*Hoxa10* Reversed	GTCTGGTGCTTCGTGTAAGGG
*Fst* Forward	TGCTGCTACTCTGCCAGTTC
*Fst* Reversed	GTGCTGCAACACTCTTCCTTG
*Il13ra2* Forward	ACCGAAATGTTGATAGCGACAG
*Il13ra2* Reversed	ACAATGCTCTGACAAATGCGTA
*Lrp2* Forward	AAAATGGAAACGGGGTGACTT
*Lrp2* Reversed	GGCTGCATACATTGGGTTTTCA
*Ihh* Forward	TCAAAGAGCTCACCCCCAAC
*Ihh* Reversed	AGTTCAGACGGTCCTTGCAG
*Pgr* Forward	TATGAGAACCCTTGACGGTGTTG
*Pgr* Reversed	CAGGGCCTGGCTCTCGTT
*Myc* Forward	TCGCTGCTGTCCTCCGAGTCC
*Myc* Reversed	GGTTTGCCTCTTCTCCACAGAC
*Elf3* Forward	TCCTCCGACTACCTTTGGCACT
*Elf3* Reversed	ACTCCAGAACCTGGGTCTTCGA
*Celsr2* Forward	CATGAAGGACCTCCAGGTGGAT
*Celsr2* Reversed	CGTTGTGGCAAATGCTGCTGTC
*Prl* Forward	CTGGCTACACCTGAAGACAAGG
*Prl* Reversed	TCACTCGAGGACTGCACCAAAC
*Prl8a2* Forward	ACCACAACCCATTCTCAGCTGG
*Prl8a2* Reversed	TGTTCAGGTCCATGAGCTGGTG
*Rrm2* Forward	TGCGAGGAGAATCTTCCAGGAC
*Rrm2* Reversed	CGATGGGAAAGACAACGAAGCG
*Ldha* Forward	ACGCAGACAAGGAGCAGTGGAA
*Ldha* Reversed	ATGCTCTCAGCCAAGTCTGCCA
*Actb* Forward	CACTGTCGAGTCGCGTCC
*Actb* Reversed	CGCAGCGATATCGTCATCCA

### RNA-seq and data analysis

The uteri of *Mettl3* cKO and control mice on GD4 were obtained for RNA-seq analysis. Total RNA was extracted from the uteri using TRIzol reagent (Invitrogen) following the manufacturer’s protocol. RNA purity and quantification were evaluated using the NanoDrop 2000 spectrophotometer (Thermo Scientific, USA). RNA integrity was assessed using the Agilent 2100 Bioanalyzer (Agilent Technologies, USA). The libraries were constructed using TruSeq Stranded mRNA LT Sample Prep Kit (Illumina, USA) and sequenced on an Illumina Novaseq6000 platform (OE Biotech Co., Ltd., Shanghai, China). The clean reads were mapped to the mouse reference genome (GRCm38.p6) using HISAT2. FPKM of each gene was calculated using Cufflinks, and the read counts of each gene were obtained by HTSeq-count. Differential expression analysis was performed using the DESeq (v3.8) R package. *p* value < 0.05 and foldchange > 1.5 or foldchange <2/3 was set as the threshold for significantly differential expression. GO, KEGG, and GSEA analyses were conducted by clusterprofiler R package (v4.4.4). All the heatmaps were drawn by pheatmap R package (version 1.0.12). Z-score were calculated and scaled by row or column.

### m^6^A sequencing and data processing

For m^6^A sequencing, the poly(A) RNA was fragmented into small pieces using Magnesium RNA Fragmentation Module (NEB, cat.e6150, USA) under 86 °C 7 min. The cleaved RNA fragments were incubated for 2 h at 4 °C with anti-m^6^A antibody (202003, Synaptic Systems) in IP buffer (50 mM Tris-HCl, 750 mM NaCl and 0.5% IGEPAL CA-630). The IP RNA was reverse-transcribed to create the cDNA by SuperScript™ II Reverse Transcriptase (1896649, Invitrogen), which was next used to synthesize U-labeled second-stranded DNAs with E. coli DNA polymerase I (m0209, NEB), RNase H (m0297, NEB) and dUTP Solution (R0133, Thermo Fisher). An A-base is then added to the blunt ends of each strand, preparing them for ligation to the indexed adapters. Each adapter contains a T-base overhang for ligating the adapter to the A-tailed fragmented DNA. Single- or dual-index adapters are ligated to the fragments, and size selection was performed with AMPureXP beads. After the heat-labile UDG enzyme (m0280, NEB) treatment of the U-labeled second-stranded DNAs, the ligated products are amplified with PCR by the following conditions: initial denaturation at 95 °C for 3 min; 8 cycles of denaturation at 98 °C for 15 s, annealing at 60 °C for 15 s, and extension at 72 °C for 30 s; and then final extension at 72 °C for 5 min. The average insert size for the final cDNA library was 300 ± 50 bp. At last, we performed the 2 × 150 bp paired-end sequencing (PE150) on an Illumina Novaseq6000 (LC-Bio Technology Co., Ltd., Hangzhou, China).

For the bioinformatic analysis of m^6^A-seq, fastp software (v0.19.4) was used to remove the reads that contained adapter contamination, low-quality bases and undetermined bases with default parameters. The sequence quality of IP and input samples were also verified using fastp. We used HISAT2 (v2.0.4) to map reads to the reference genome of mouse (GRCm38.p6). Mapped reads of IP and input libraries were provided for R package exomePeak (v2.13), which identifies m^6^A peaks. The bigwig format was converted from the bam format by bamcoverage tool in deeptools (version 2.5.4), and was adapted for visualization on the IGV software (v2.14.0). RPGC (reads per genomic content) method was used for normalization. MEME (v4.12.0) and HOMER (v4.10) were used for de novo and known motif finding followed by localization of the motif with respect to peak summit. Called peaks were annotated by intersection with gene architecture using R package ChIPseeker (v3.8). Then StringTie (v2.1.2) was used to perform expression level for all mRNAs from input libraries by calculating FPKM (total exon fragments/mapped reads (millions) × exon length (kB)). The differentially expressed mRNAs were selected with log_2_(fold change) >1 or log_2_(fold change) <−1 and *P* < 0.05 by R package edgeR (v3.38.4).

### m^6^A-RIP-qPCR

Purified mRNAs of the uteri of *Mettl3* cKO mice and control mice were prepared and fragmented into ~100 nt by RNA fragmentation reagents (e6150, NEB). Immunoprecipitation was performed using anti-m^6^A antibody (202003, Synaptic Systems) as described previously. The enrichment of m^6^A was measured with quantitative RT-PCR. Primers for m^6^A-RIP-qPCR are listed in Table 2.

**Table 2:** List of primer sequences for m^6^A-RIP-qPCR.

Primer name	Sequences(5′-3′)
*Pgr* Forward	TAGAGCAACCTGCAACCAGAA
*Pgr* Reversed	AGCCCATTCTTACTCGTTCTCC
*Myc* Forward	AACGACGAGAACAGTTGAAACAC
*Myc* Reversed	AGCTCCTCCTCGAGTTAGGTC
*Elf3* Forward	AATTAAGGATCGGGGCTGGAC
*Elf3* Reversed	GCAACACAGGGAACACATCC
*Celsr2* Forward	CTCTCCCAGGAACTGACAAGC
*Celsr2* Reversed	AAACGGTTCATGCAGCATTTGG

### mRNA stability assay

To assess mRNA stability, primary uterine cells were treated with actinomycin D (Sigma) at a final concentration of 5 μg/mL for 0 h, 2 h, and 4 h. Total RNA samples were extracted and subjected to RT-qPCR analysis. Results were normalized to the expression of *Actb*. Fold differences in expression levels were calculated according to the 2^−ΔΔCT^ method.

### Statistics

Statistical analyses were performed using 2-tailed Student’s *t* test. Data are presented as mean ± SD. *P* values less than 0.05 were considered statistically significant. *P < 0.05, **P < 0.01 and ***P < 0.001.

## Supplementary information

Reproducibility checklist

Supplementary Figures and Table

Original Data File-Western blot results

## Data Availability

The RNA-seq data were deposited in the GEO repository at NCBI under the accession number GSE208096. The m^6^A-seq data were available from the corresponding author upon request. The code used for GEO data process is available at https://github.com/wansh007/data-process.

## References

[1] Agarwal A, Mulgund A, Hamada A, Chyatte MR (2015). A unique view on male infertility around the globe. Reprod Biol Endocrinol.

[2] Zondervan KT, Becker CM, Missmer SA (2020). Endometriosis. N Engl J Med.

[3] Kuivasaari P, Hippeläinen M, Anttila M, Heinonen S (2005). Effect of endometriosis on IVF/ICSI outcome: stage III/IV endometriosis worsens cumulative pregnancy and live-born rates. Hum Reprod.

[4] Barnhart K, Dunsmoor-Su R, Coutifaris C (2002). Effect of endometriosis on in vitro fertilization. Fertil Steril.

[5] Pantos K, Grigoriadis S, Maziotis E, Pistola K, Xystra P, Pantou A, et al. The role of interleukins in recurrent implantation failure: a comprehensive review of the literature. Int J Mol Sci. 2022,23. 10.3390/ijms23042198 10.3390/ijms23042198 PMC8875813 35216313

[6] Izawa M, Taniguchi F, Terakawa N, Harada T (2013). Epigenetic aberration of gene expression in endometriosis. Front Biosci (Elite Ed).

[7] Kim TH, Yoo JY, Choi KC, Shin JH, Leach RE, Fazleabas AT, et al. Loss of HDAC3 results in nonreceptive endometrium and female infertility. Sci Transl Med. 2019, 11. 10.1126/scitranslmed.aaf7533 10.1126/scitranslmed.aaf7533 PMC6650287 30626716

[8] Xu H, Zhou M, Cao Y, Zhang D, Han M, Gao X (2019). Genome-wide analysis of long noncoding RNAs, microRNAs, and mRNAs forming a competing endogenous RNA network in repeated implantation failure. Gene.

[9] Ghafouri-Fard S, Shoorei H, Taheri M (2020). Role of non-coding RNAs in the pathogenesis of endometriosis. Front Oncol.

[10] Murakami S, Jaffrey SR (2022). Hidden codes in mRNA: control of gene expression by m(6)A. Mol Cell.

[11] Zaccara S, Ries RJ, Jaffrey SR (2019). Reading, writing and erasing mRNA methylation. Nat Rev Mol cell Biol.

[12] Xu K, Yang Y, Feng GH, Sun BF, Chen JQ, Li YF (2017). Mettl3-mediated m(6)A regulates spermatogonial differentiation and meiosis initiation. Cell Res.

[13] Mu H, Li H, Liu Y, Wang X, Mei Q, Xiang W (2022). N6-Methyladenosine modifications in the female reproductive system: roles in gonad development and diseases. Int J Biol Sci.

[14] Liu J, Eckert MA, Harada BT, Liu SM, Lu Z, Yu K (2018). m(6)A mRNA methylation regulates AKT activity to promote the proliferation and tumorigenicity of endometrial cancer. Nat Cell Biol.

[15] Zhai J, Li S, Sen S, Opoku-Anane J, Du Y, Chen ZJ (2020). m(6)A RNA methylation regulators contribute to eutopic endometrium and myometrium dysfunction in adenomyosis. Front Genet.

[16] Jiang L, Zhang M, Wu J, Wang S, Yang X, Yi M (2020). Exploring diagnostic m6A regulators in endometriosis. Aging.

[17] Li X, Xiong W, Long X, Dai X, Peng Y, Xu Y (2021). Inhibition of METTL3/m6A/miR126 promotes the migration and invasion of endometrial stromal cells in endometriosis. Biol Reprod.

[18] Soyal SM, Mukherjee A, Lee KY, Li J, Li H, DeMayo FJ (2005). Cre-mediated recombination in cell lineages that express the progesterone receptor. Genes.

[19] Wang H, Dey SK (2006). Roadmap to embryo implantation: clues from mouse models. Nat Rev Genet.

[20] Wetendorf M, DeMayo FJ (2012). The progesterone receptor regulates implantation, decidualization, and glandular development via a complex paracrine signaling network. Mol Cell Endocrinol.

[21] Wang X, Wu SP, DeMayo FJ (2017). Hormone dependent uterine epithelial-stromal communication for pregnancy support. Placenta.

[22] Marquardt RM, Kim TH, Shin JH, Jeong JW. Progesterone and estrogen signaling in the endometrium: what goes wrong in endometriosis? Int J Mol Sci. 2019,20. 10.3390/ijms20153822 10.3390/ijms20153822 PMC6695957 31387263

[23] Rubel CA, Lanz RB, Kommagani R, Franco HL, Lydon JP, DeMayo FJ (2012). Research resource: genome-wide profiling of progesterone receptor binding in the mouse uterus. Mol Endocrinol.

[24] Huet-Hudson YM, Andrews GK, Dey SK (1989). Cell type-specific localization of c-myc protein in the mouse uterus: modulation by steroid hormones and analysis of the periimplantation period. Endocrinology.

[25] Li SY, Yan JQ, Song Z, Liu YF, Song MJ, Qin JW (2017). Molecular characterization of lysyl oxidase-mediated extracellular matrix remodeling during mouse decidualization. FEBS Lett.

[26] Lei W, Feng XH, Deng WB, Ni H, Zhang ZR, Jia B (2012). Progesterone and DNA damage encourage uterine cell proliferation and decidualization through up-regulating ribonucleotide reductase 2 expression during early pregnancy in mice. J Biol Chem.

[27] Zeller KI, Jegga AG, Aronow BJ, O’Donnell KA, Dang CV (2003). An integrated database of genes responsive to the Myc oncogenic transcription factor: identification of direct genomic targets. Genome Biol.

[28] Liberzon A, Subramanian A, Pinchback R, Thorvaldsdóttir H, Tamayo P, Mesirov JP (2011). Molecular signatures database (MSigDB) 3.0. Bioinformation.

[29] Zuo RJ, Gu XW, Qi QR, Wang TS, Zhao XY, Liu JL (2015). Warburg-like glycolysis and lactate shuttle in mouse decidua during early pregnancy. J Biol Chem.

[30] Zhao S, Lu J, Chen Y, Wang Z, Cao J, Dong Y (2021). Exploration of the potential roles of m6A regulators in the uterus in pregnancy and infertility. J Reprod Immunol.

[31] Zhang Z, Zhou D, Lai Y, Liu Y, Tao X, Wang Q (2012). Estrogen induces endometrial cancer cell proliferation and invasion by regulating the fat mass and obesity-associated gene via PI3K/AKT and MAPK signaling pathways. Cancer Lett.

[32] Vasquez YM, DeMayo FJ (2013). Role of nuclear receptors in blastocyst implantation. Semin Cell Dev Biol.

[33] Large MJ, DeMayo FJ (2012). The regulation of embryo implantation and endometrial decidualization by progesterone receptor signaling. Mol Cell Endocrinol.

[34] Rubel CA, Jeong JW, Tsai SY, Lydon JP, Demayo FJ (2010). Epithelial-stromal interaction and progesterone receptors in the mouse uterus. Semin Reprod Med.

[35] Kim TH, Yoo JY, Wang Z, Lydon JP, Khatri S, Hawkins SM (2015). ARID1A is essential for endometrial function during early pregnancy. PLoS Genet.

[36] Kurihara I, Lee DK, Petit FG, Jeong J, Lee K, Lydon JP (2007). COUP-TFII mediates progesterone regulation of uterine implantation by controlling ER activity. PLoS Genet.

[37] Lessey BA, Kim JJ (2017). Endometrial receptivity in the eutopic endometrium of women with endometriosis: it is affected, and let me show you why. Fertil Steril.

[38] Yin X, Pavone ME, Lu Z, Wei J, Kim JJ (2012). Increased activation of the PI3K/AKT pathway compromises decidualization of stromal cells from endometriosis. J Clin Endocrinol Metab.

[39] Gajulapalli VN, Samanthapudi VS, Pulaganti M, Khumukcham SS, Malisetty VL, Guruprasad L (2016). A transcriptional repressive role for epithelial-specific ETS factor ELF3 on oestrogen receptor alpha in breast cancer cells. Biochem J.

[40] Qiao Z, Jiang Y, Wang L, Wang L, Jiang J, Zhang J (2019). Mutations in KIAA1109, CACNA1C, BSN, AKAP13, CELSR2, and HELZ2 are associated with the prognosis in endometrial cancer. Front Genet.

[41] Tan J, Che Y, Liu Y, Hu J, Wang W, Hu L (2021). CELSR2 deficiency suppresses lipid accumulation in hepatocyte by impairing the UPR and elevating ROS level. FASEB J.

[42] Xu M, Zhu S, Xu R, Lin N (2020). Identification of CELSR2 as a novel prognostic biomarker for hepatocellular carcinoma. BMC Cancer.

[43] Zhou X, Zhan Z, Tang C, Li J, Zheng X, Zhu S (2020). Silencing Celsr2 inhibits the proliferation and migration of Schwann cells through suppressing the Wnt/β-catenin signaling pathway. Biochem Biophys Res Commun.

[44] Gao Y, Ouyang X, Zuo L, Xiao Y, Sun Y, Chang C (2021). R-2HG downregulates ERα to inhibit cholangiocarcinoma via the FTO/m6A-methylated ERα/miR16-5p/YAP1 signal pathway. Mol Ther Oncol.

[45] Chen Z, Wu L, Zhou J, Lin X, Peng Y, Ge L (2020). N6-methyladenosine-induced ERRγ triggers chemoresistance of cancer cells through upregulation of ABCB1 and metabolic reprogramming. Theranostics.

[46] Kurita T, Lee K-j, Saunders PTK, Cooke PS, Taylor JA, Lubahn DB (2001). Regulation of progesterone receptors and decidualization in uterine stroma of the estrogen receptor-α knockout mouse1. Biol Reprod.

[47] Tan J, Paria BC, Dey SK, Das SK (1999). Differential uterine expression of estrogen and progesterone receptors correlates with uterine preparation for implantation and decidualization in the mouse. Endocrinology.

[48] Lee K, Jeong J, Kwak I, Yu CT, Lanske B, Soegiarto DW (2006). Indian hedgehog is a major mediator of progesterone signaling in the mouse uterus. Nat Genet.

[49] Tranguch S, Cheung-Flynn J, Daikoku T, Prapapanich V, Cox MB, Xie H (2005). Cochaperone immunophilin FKBP52 is critical to uterine receptivity for embryo implantation. Proc Natl Acad Sci USA.

[50] Gu Y, Zhang X, Yang Q, Wang JM, He YP, Sun ZG (2015). Uterine NDRG2 expression is increased at implantation sites during early pregnancy in mice, and its down-regulation inhibits decidualization of mouse endometrial stromal cells. Reprod Biol Endocrinol.

[51] Yang Q, Gu Y, Zhang X, Wang JM, He YP, Shi Y (2016). Uterine expression of NDRG4 is induced by estrogen and up-regulated during embryo implantation process in mice. PloS One.

[52] Chen H, Liu H, Qing G (2018). Targeting oncogenic Myc as a strategy for cancer treatment. Signal Transduct Target Ther.

[53] Wan S, Sun Y, Fu J, Song H, Xiao Z, Yang Q (2022). mTORC1 signaling pathway integrates estrogen and growth factor to coordinate vaginal epithelial cells proliferation and differentiation. Cell Death Dis.

[54] McLean AC, Valenzuela N, Fai S, Bennett SA. Performing vaginal lavage, crystal violet staining, and vaginal cytological evaluation for mouse estrous cycle staging identification. *J Vis Exp.* 2012:e4389. 10.3791/4389 10.3791/4389 PMC3490233 23007862

[55] Fullerton PT, Monsivais D, Kommagani R, Matzuk MM (2017). Follistatin is critical for mouse uterine receptivity and decidualization. Proc Natl Acad Sci USA.

[56] Lydon JP, DeMayo FJ, Funk CR, Mani SK, Hughes AR, Montgomery CA (1995). Mice lacking progesterone receptor exhibit pleiotropic reproductive abnormalities. Genes Dev.

[57] Liang X-H, Deng W-B, Li M, Zhao Z-A, Wang T-S, Feng X-H (2014). Egr1 protein acts downstream of estrogen-leukemia inhibitory factor (LIF)-STAT3 pathway and plays a role during implantation through targeting Wnt4*. J Biol Chem.

