# Supplementary material for: METTL3-dependent m6A methylation facilitates uterine receptivity and female fertility via balancing estrogen and progesterone signaling
Source: Cell Death Dis. 2023 Jun 3;14(6):349. doi: 10.1038/s41419-023-05866-1 (PMC10239469; doi:10.1038/s41419-023-05866-1)
Supplement: Supplementary file 2 — Supplementary Figures and Table [file 41419_2023_5866_MOESM2_ESM.docx]

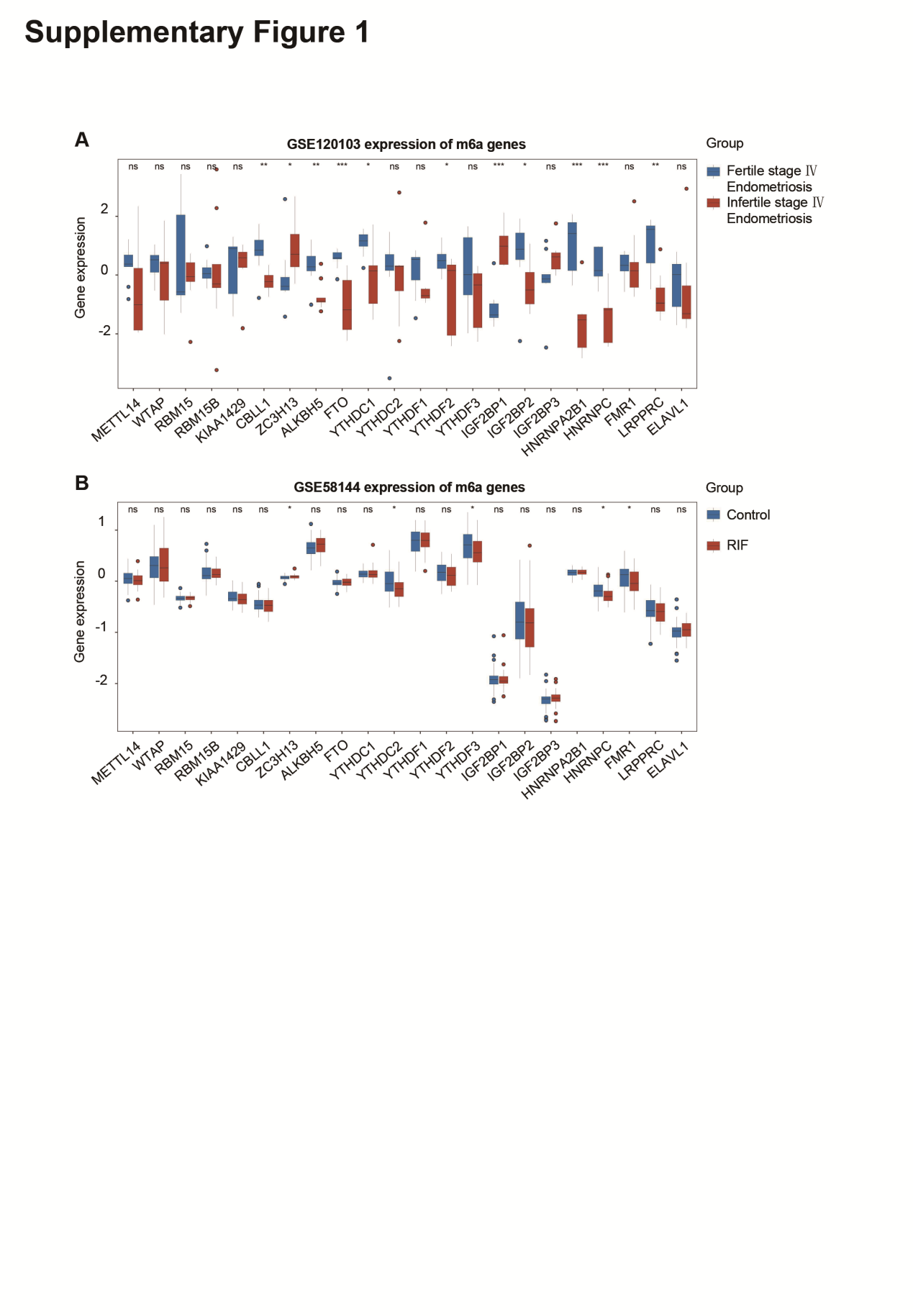


**Supplementary Fig. 1. Expression landscape of m^6^A regulators in the endometrium of infertile women with endometriosis or recurrent implantation failure. A** Expression landscape of m^6^A regulators in the endometrium of fertile (n=9) and infertile (n=9) women with stage IV endometriosis in dataset GSE120103. Data are presented as mean ± SD, **P* < 0.05, ***P* < 0.01, ****P* < 0.001, relative to “Fertile stage Ⅳ Endometriosis” group. **B** Expression landscape of m^6^A regulators in the endometrium of women with RIF following in vitro fertilization (IVF) treatment (n=43) and healthy control women (n=72) 7 days after the putative luteinizing hormone surge (GSE58144). Data are presented as mean ± SD, **P* < 0.05, relative to control.


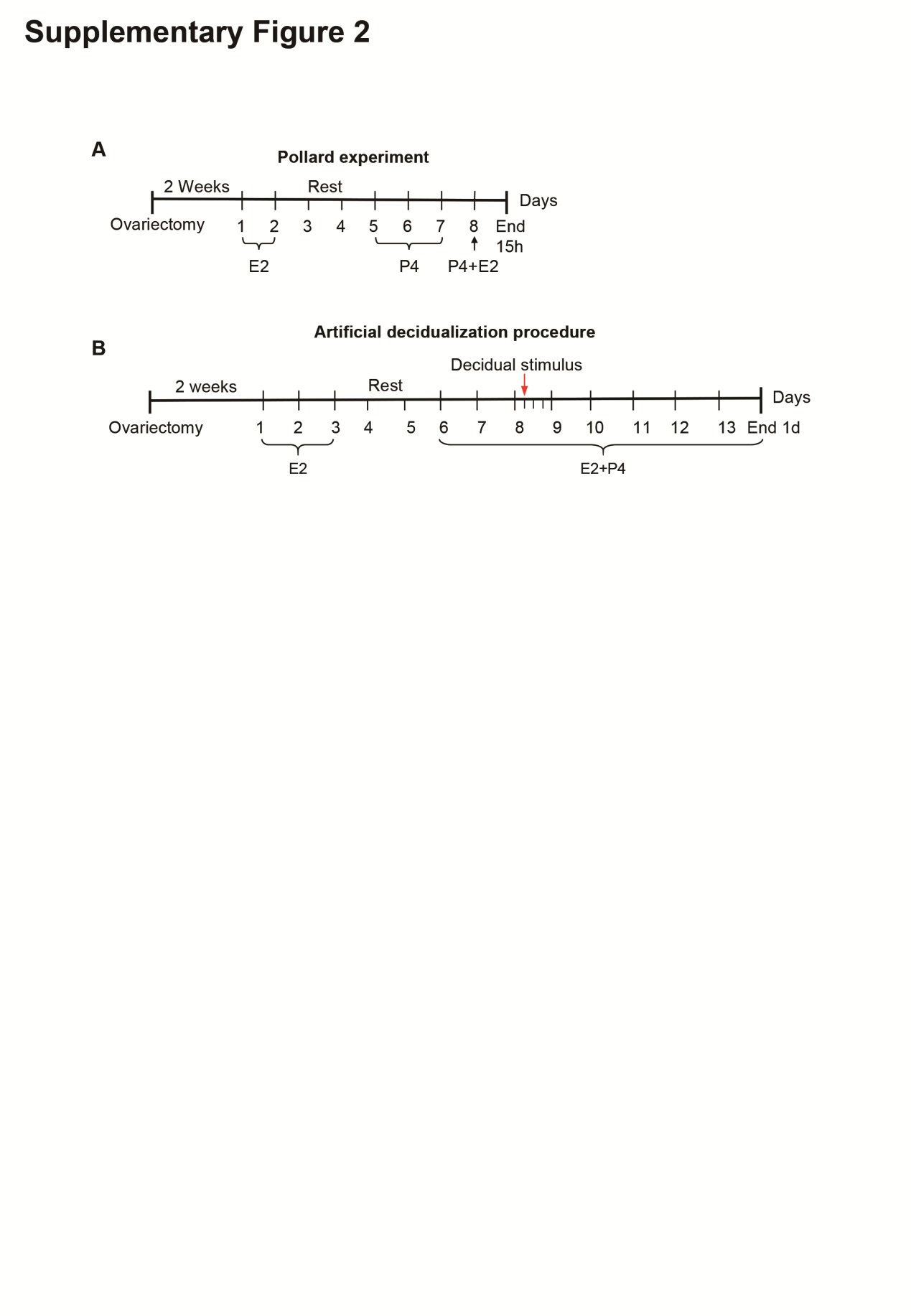


## **Supplementary Fig. 2. Experimental schemes of in vivo artificial pregnancy or decidual response. A** Experimental scheme used to induce artificial pregnancy in *Mettl3* cKO and control mice (pollard experiment). **B** Experimental scheme of in vivo artificial decidual response.


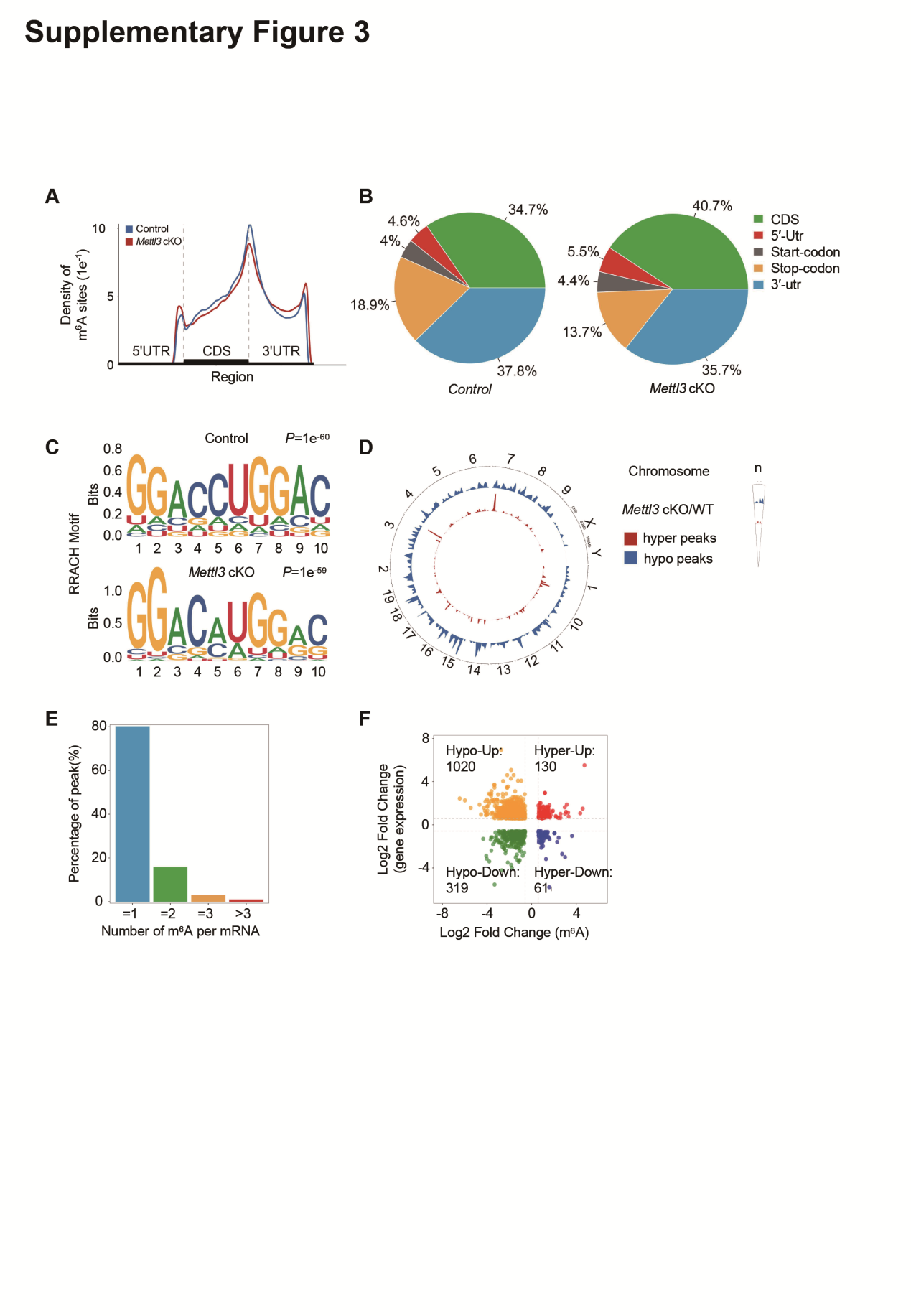


## **Supplementary Fig. 3. Global m^6^A profiles in the uterus of *Mettl3* cKO and control mice by m^6^A-seq. A** Metagene profile of m^6^A site distribution along a normalized transcript containing three rescaled non-overlapping segments: 5′UTR, CDS, and 3′UTR in the uterus of *Mettl3* cKO and control mice. **B** Pie chart showing the distribution of m^6^A sites in five regions of the uterus of *Mettl3* cKO and control mice. **C** RRACH motif analysis revealed the top consensus m^6^A motif in the uterus of *Mettl3* cKO and control mice. **D** Circos plot showing the distribution of hypermethylated (hyper) and hypomethylated (hypo) m^6^A peaks in the transcriptome of the uterus of *Mettl3* cKO mice compared with that of control mice. **E** Bar chart depicting the percentage of mRNAs with different internal m^6^A abundance. **F** Distribution of genes with a significant change in both m^6^A level and gene expression level in the uterus of *Mettl3* cKO mice and control mice (fold-change = 1.5 and *P* value = 0.05).

**
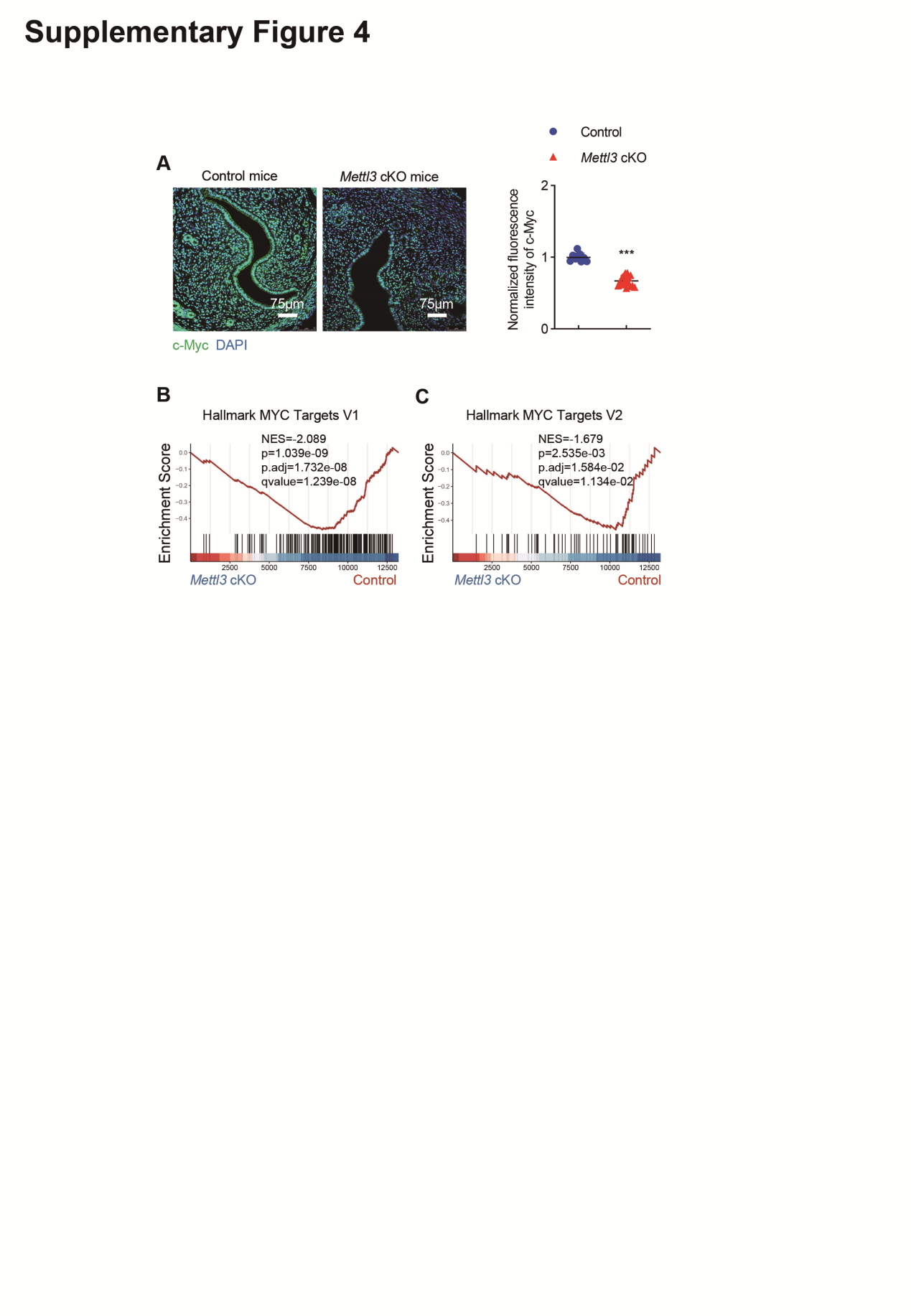
**

## **Supplementary Fig. 4. Uterine c-Myc level in *Mettl3* cKO and control females. A** Representative immunofluorescence images and quantification of c-Myc in the uterus of *Mettl3* cKO and control mice following induction of artificial pregnancy (pollard experiment) in Fig. S2A. Nuclei were stained with DAPI. Scale bars: 75 μm. Fluorescence intensities of uterine c-Myc were calculated using 9 images from 3 control mice and 16 images from 3 *Mettl3* cKO mice. Results are representative of 3 independent experiments. Data are presented as mean ± SD. ****P* < 0.001. **B, C** Gene set enrichment analysis of transcriptomic changes of the uterus in *Mettl3* cKO mice relative to control mice, using Hallmark gene sets “Hallmark MYC Targets V1” (**B**) and “Hallmark MYC Targets V2” (**C**).
