# Supplementary figures and images for: METTL3-dependent m6A methylation facilitates uterine receptivity and female fertility via balancing estrogen and progesterone signaling
Source: Cell Death Dis. 2023 Jun 3;14(6):349. doi: 10.1038/s41419-023-05866-1 (PMC10239469; doi:10.1038/s41419-023-05866-1)

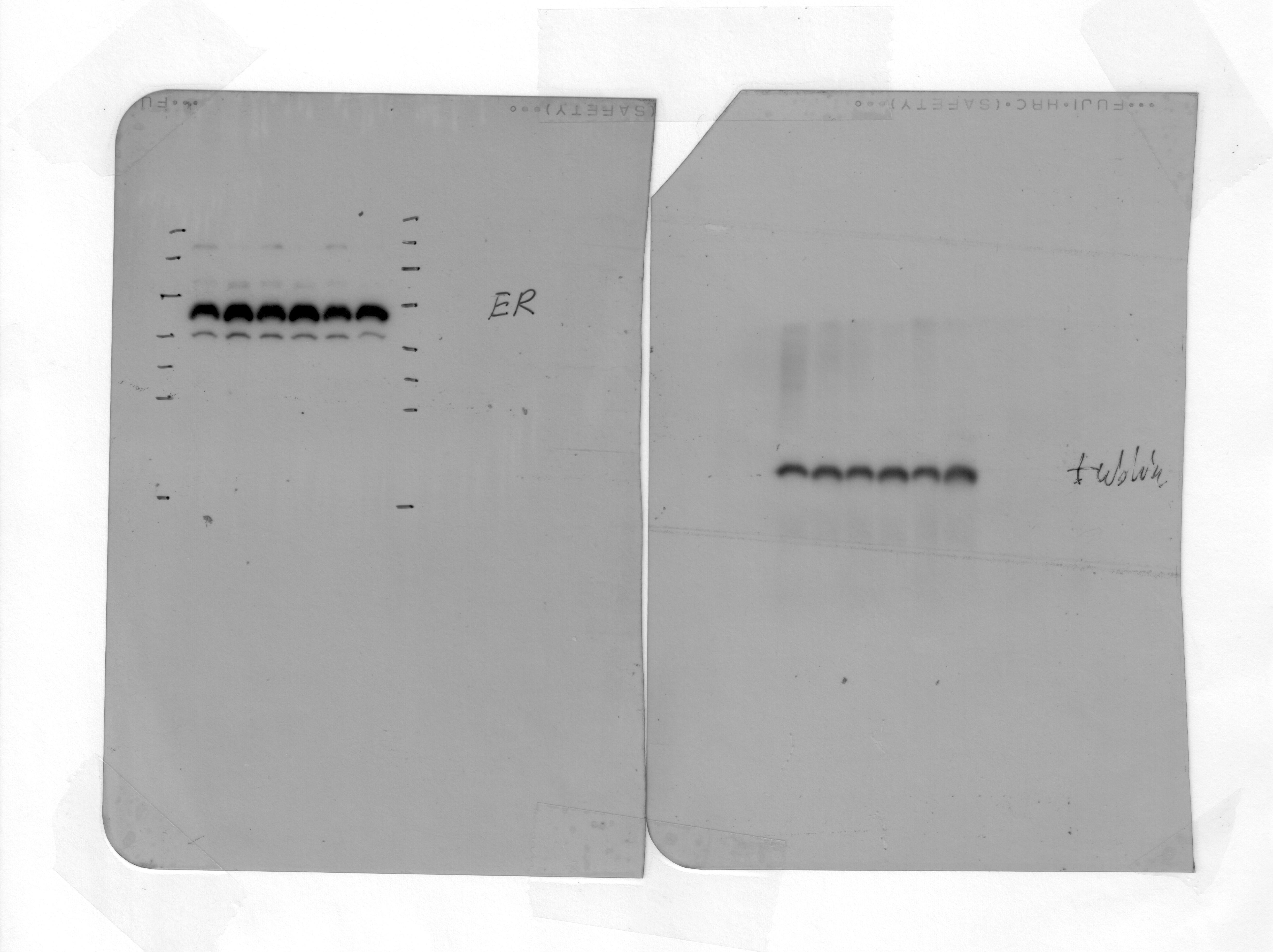

Supplement: Supplementary file 3 — Original Data File-Western blot results [file 41419_2023_5866_MOESM3_ESM.tif]
